# Supplementary material for: Transcriptome analysis of two isolates of the tomato pathogen Cladosporium fulvum, uncovers genome-wide patterns of alternative splicing during a host infection cycle
Source: PLoS Pathog. 2024 Dec 18;20(12):e1012791. doi: 10.1371/journal.ppat.1012791 (PMC11694984; doi:10.1371/journal.ppat.1012791)
Supplement: S1 Text — (PDF) [file ppat.1012791.s001.pdf]

Supplementary Results

S1 Text. RNA sequencing of *C. fulvum* isolates Race 5 and Race 4 during interaction with tomato.

The transcriptomes of *C. fulvum* isolates Race 5 and Race 4 were sequenced at high depth during their interaction with *Solanum lycopersicum* cv. Moneymaker at seven timepoints (2, 4, 6, 8, 10, 12, and 14 dpi) and from three independent infections (i.e. biological replicates), generating between 65 M and 221 M RNAseq reads per sample (S1 Table). As fungal biomass during the early stages of the infection is typically minimal, RNAseq libraries produced from infected tissue samples collected at 2, 4, 6, and 8 dpi were sequenced at higher depths compared to RNAseq libraries corresponding to samples from 10, 12, and 14 dpi (S1 Table), thereby increasing the chances of capturing fungal transcripts from early in the infection process. Before RNAseq read mapping, reads were assigned to either the *C. fulvum* isolate Race 5 [1] (Zaccaron et al., 2022) or the tomato reference genome of cv. Heinz 1706 version SL4.0 [2] (Hosmani et al., 2019), using the alignment-free *k*-mer method implemented in the *seal.sh* script from the BBMap package (S2 Table). Reads matching the tomato genome were removed and the remaining reads were mapped to the *C. fulvum* genome. As expected, at early timepoints, the percentage of reads mapping to the *C. fulvum* genome was considerably low (Table 1). Specifically, at 2 dpi and 4 dpi, between 135,953 (0.08%) and 437,311 (0.21%) of the reads obtained from tomato leaf samples infected with *C. fulvum* Race 5, and between 409,880 (0.23%) and 1,852,149 (0.92%) of the reads from tomato leaf samples infected with *C. fulvum* Race 4 mapped to the *C. fulvum* Race 5 genome. However, the percentage of reads that successfully mapped to the *C. fulvum* genome significantly increased from samples collected at later timepoints, reaching between 27.1 M (32.55%) and 37.6 M (43.87%) for isolate Race 5, and between 12.7 M (19.5%) and 45.7 M (57.64%) reads for isolate Race 4 at 12 dpi and 14 dpi, respectively (Table 1).

References

1. Zaccaron, A.Z., Chen, L.-H., Samaras, A., Stergiopoulos, I., 2022. A chromosome-scale genome assembly of the tomato pathogen Cladosporium fulvum reveals a compartmentalized genome architecture and the presence of a dispensable chromosome. Microb. Genomics 8, 000819. https://doi.org/10.1099/mgen.0.000819
2. Hosmani, P.S., Flores-Gonzalez, M., van de Geest, H., Maumus, F., Bakker, L.V., Schijlen, E., van Haarst, J., Cordewener, J., Sanchez-Perez, G., Peters, S., Fei, Z., Giovannoni, J.J., Mueller, L.A., Saha, S., 2019. An improved de novo assembly and annotation of the tomato reference genome using single-molecule sequencing, Hi-C proximity ligation and optical maps. bioRxiv 767764. https://doi.org/10.1101/767764
