# Supplementary material for: Transcriptome analysis of two isolates of the tomato pathogen Cladosporium fulvum, uncovers genome-wide patterns of alternative splicing during a host infection cycle
Source: PLoS Pathog. 2024 Dec 18;20(12):e1012791. doi: 10.1371/journal.ppat.1012791 (PMC11694984; doi:10.1371/journal.ppat.1012791)
Supplement: S2 Text — (PDF) [file ppat.1012791.s002.pdf]

Supplementary Results

S2 Text. Isolates Race 5 and Race 4 of *C. fulvum* share nearly all their intron splice sites.

Intron-containing genes may undergo alternative splicing (AS), thereby generating diverse mRNA isoforms. A total 9,356 and 9,278 intron-containing genes have been reported in the genomes of isolates Race 5 and Race 4, respectively [1] (Zaccaron and Stergiopoulos, 2024) (S3 Table). As introns are spliced by spliceosomes at conserved sequences, a splice site analysis was first conducted to determine to what extent intron splice sites were conserved between orthologous genes in the two isolates. To start the analysis, it was first determined whether the introns that were predicted in the genes of *C. fulvum* isolates Race 5 and Race 4 [1] (Zaccaron and Stergiopoulos, 2024) were supported by the RNAseq data generated in this study, by mapping the reads to the genomes of the two isolates. Ensuing splice site analysis revealed that from the 17,029 and 16,842 introns predicted in the genes of isolates Race 5 and Race 4, 13,639 (80.1%) and 12,718 (75.5%) could be confirmed with at least five RNAseq reads, respectively. Moreover, from the 9,356 and 9,278 intron-containing genes in isolates Race 5 and Race 4, 7,769 (83.0%) and 7,284 (78.5%) had at least one of their introns confirmed, and 5,309 (56.7%) and 4,453 (48.0%) had all their introns confirmed, respectively. This indicated that the predicted exon-intron structures in the intron-containing genes of isolates Race 5 and Race 4 were well-supported by the RNAseq data.

To further investigate whether orthologous genes in isolates Race 5 and Race 4 had similar number and size of introns, a total of 14,747 one-to-one ortholog gene pairs were analyzed. These ortholog pairs were obtained with OrthoFinder [2] (Emms and Kelly, 2015), and included 98.4% and 99.0% of all predicted genes in isolates Race 5 (*n*= 14,993) and Race 4 (*n*= 14,895), respectively. From the 14,747 ortholog gene pairs, 14,739 (99.9%) pairs had the same number of introns in both orthologous genes (Fig 1A) and 14,648 (99.3%) pairs had the same total size of intronic sequences (Fig 1B), thereby indicating a high conservation in the number and size of introns between the two isolates.

Finally, we investigated whether orthologous genes between the two isolates also shared the same start and end coordinates of their introns. To do so, the gene annotation of isolate Race 4 was mapped to the genome of isolate Race 5 using Liftoff, thereby resulting in a new annotation for isolate Race 4 but with its gene coordinates now modeled on the genome of Race 5. This enabled intron coordinates to be compared between pairs of ortholog genes, which revealed that from the 14,747 one-to-one gene orthologs between isolate Race 5 and Race 4, 14,729 (99.9%) had the same number of introns with the same start and end coordinates, and only 18 ortholog pairs had different number of introns or intron coordinates. These results indicated that splicing sites and intron coordinates are highly conserved between the two *C. fulvum* isolates.

References

1. Zaccaron, A.Z., Stergiopoulos, I., 2024. Analysis of five near-complete genome assemblies of the tomato pathogen Cladosporium fulvum uncovers additional accessory chromosomes and structural variations induced by transposable elements effecting the loss of avirulence genes. BMC Biol. 22, 25. https://doi.org/10.1186/s12915-024-01818-z
2. Emms, D.M., Kelly, S., 2015. OrthoFinder: solving fundamental biases in whole genome comparisons dramatically improves orthogroup inference accuracy. Genome Biol. 16, 1–14.
