# Supplementary material for: Transcriptome analysis of two isolates of the tomato pathogen Cladosporium fulvum, uncovers genome-wide patterns of alternative splicing during a host infection cycle
Source: PLoS Pathog. 2024 Dec 18;20(12):e1012791. doi: 10.1371/journal.ppat.1012791 (PMC11694984; doi:10.1371/journal.ppat.1012791)
Supplement: S3 Text — (PDF) [file ppat.1012791.s003.pdf]

Supplementary Results

S3 Text. Transcriptome profiling of *C. fulvum* during host infections reveals extensive transcript isoform heterogeneity among isolates and infections.

The RNAseq reads that were obtained in this study for the two isolates were then used to perform reference-based transcriptome assemblies, using the genome of isolate Race 5 [1] (Zaccaron et al., 2022) as reference. However, preliminary attempts indicated the presence of chimeric transcripts in the data, which possibly resulted from overlapping untranslated regions of physically close neighboring genes (S1 Fig). Therefore, transcripts were instead assembled in a gene-by-gene strategy, in which reads that mapped to the genes of each isolate were first extracted and then used to assemble the unique transcript isoforms that originated from each individual gene. Using these reads, a total of 612,075 transcripts could be assembled from reads mapping to the genes of the two isolates across all timepoints and infection replicates (S4 Table). However, many of the transcripts were assembled multiple times among the samples, indicating that they were redundant. To obtain a set of unique transcripts, all the 612,075 assembled transcripts were next clustered such that identical or fully contained sequences (i.e. a sequence within a sequence) were clustered together, and the longest sequence from each cluster was chosen as the representative. In doing so, a total of 57,148 clusters were generated, each of which represented a putative unique transcript isoform.

References

1. Zaccaron, A.Z., Chen, L.-H., Samaras, A., Stergiopoulos, I., 2022. A chromosome-scale genome assembly of the tomato pathogen Cladosporium fulvum reveals a compartmentalized genome architecture and the presence of a dispensable chromosome. Microb. Genomics 8, 000819. https://doi.org/10.1099/mgen.0.000819
