# Supplementary material for: Transcriptome analysis of two isolates of the tomato pathogen Cladosporium fulvum, uncovers genome-wide patterns of alternative splicing during a host infection cycle
Source: PLoS Pathog. 2024 Dec 18;20(12):e1012791. doi: 10.1371/journal.ppat.1012791 (PMC11694984; doi:10.1371/journal.ppat.1012791)
Supplement: S1 Fig — (PDF) [file ppat.1012791.s004.pdf]

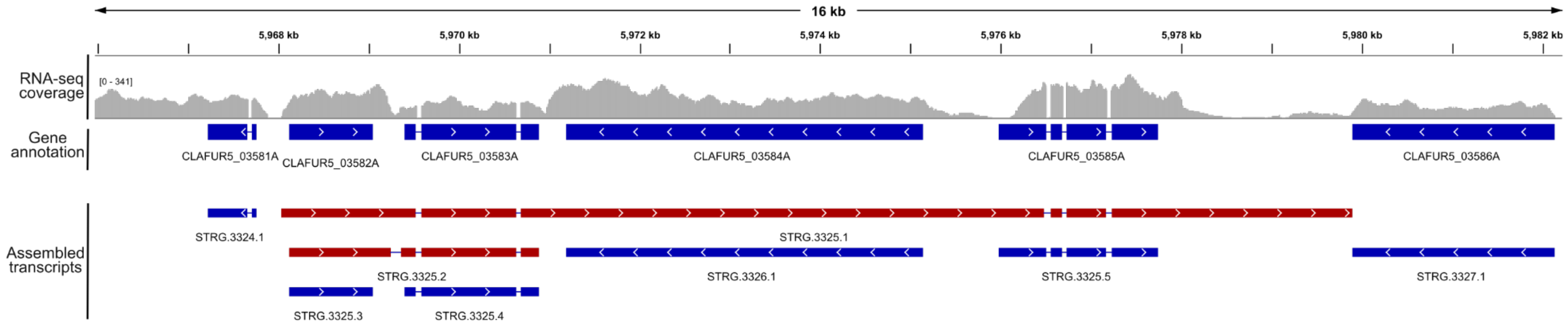

S1 Fig. Preliminary transcriptome assembly generated chimeric transcripts spanning genes physically close in the genome. The figure shows a region of 16 kb in chromosome 1 of *Cladosporium fulvum* isolate Race 5 containing six predicted genes. RNA-seq red coverage from the first of the three infections that were performed (i.e. biological replicate 1) at 14 dpi during interaction with tomato is shown above the predicted genes. The reference-based transcriptome assembly based on the reads shown in the figure resulted in chimeric transcripts (in red), that span multiple genes.
