# Supplementary material for: Transcriptome analysis of two isolates of the tomato pathogen Cladosporium fulvum, uncovers genome-wide patterns of alternative splicing during a host infection cycle
Source: PLoS Pathog. 2024 Dec 18;20(12):e1012791. doi: 10.1371/journal.ppat.1012791 (PMC11694984; doi:10.1371/journal.ppat.1012791)
Supplement: S2 Fig — (PDF) [file ppat.1012791.s005.pdf]

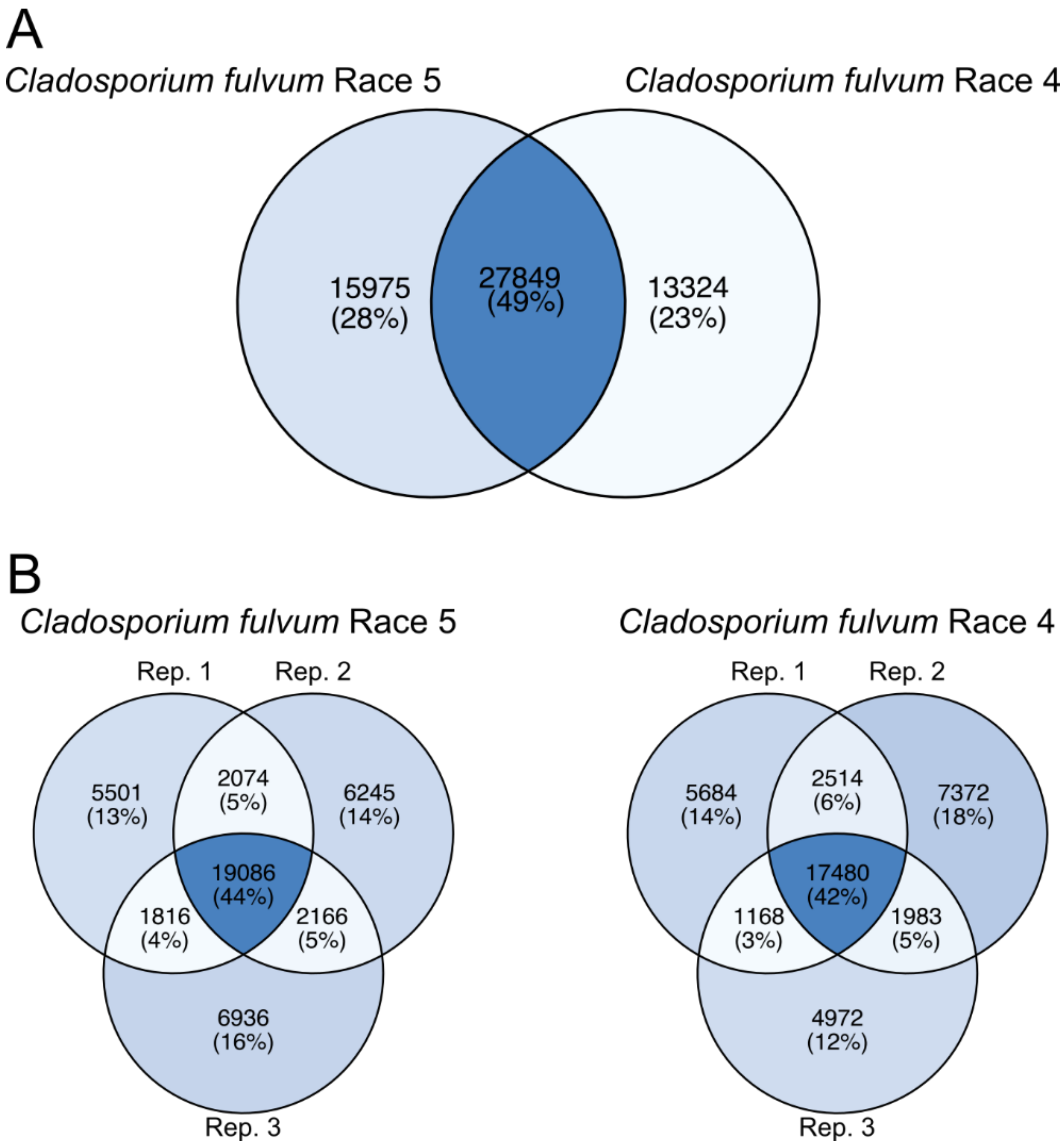

S2 Fig. A high heterogeneity in transcripts produced by *Cladosporium fulvum* isolates Race 5 and Race 4 during tomato infections is seen between the two isolates and the three different infections that were performed with each isolate. (A) The total number of transcripts that are shared between isolates Race 5 and Race 4. The Venn diagram shows all uniquely assembled transcripts, after combining all transcripts assembled across three independent tomato infections (i.e. biological replicates) and the seven timepoints sampled in each infection. (B) The total number of assembled transcripts shared by all biological replicates (Rep. 1, Rep. 2, and Rep. 3) for isolates Race 5 and Race 4. The Venn diagrams show all unique transcripts assembled for each infection and isolate, after combining the assembled transcripts from all seven sampled timepoints during the infection. Darker colors of intersections indicate higher numbers.
