# Supplementary material for: Transcriptome analysis of two isolates of the tomato pathogen Cladosporium fulvum, uncovers genome-wide patterns of alternative splicing during a host infection cycle
Source: PLoS Pathog. 2024 Dec 18;20(12):e1012791. doi: 10.1371/journal.ppat.1012791 (PMC11694984; doi:10.1371/journal.ppat.1012791)
Supplement: S3 Fig — (PDF) [file ppat.1012791.s006.pdf]

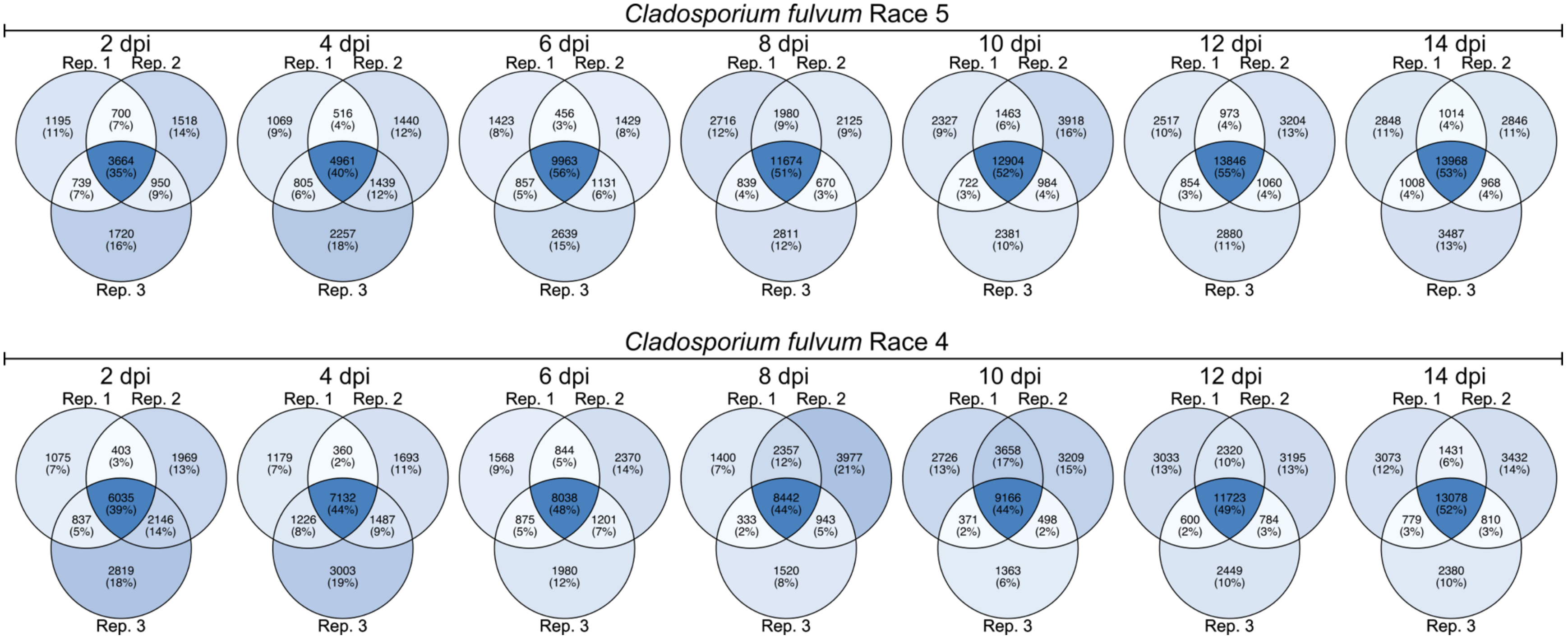

S3 Fig. An overall low number of transcripts are constitutively present in samples from all three tomato infections (i.e. biological replicates) performed either with *Cladosporium fulvum* isolate Race 5 or isolate Race 4, and in every of the seven infection timepoints that were sampled per infection. Venn diagrams showing the number of transcripts supported by one, two, or all three biological replicates (Rep. 1, Rep. 2, and Rep. 3) at each sampled infection timepoint (2, 4, 6, 8, 10, 12, 14 dpi) for isolates Race 5 and Race 4. A biological replicate represents a different infection experiment with isolates Race 5 and Race 4. Darker colors of intersections indicate higher numbers. To identify transcripts supported by each replicate, all assembled transcripts were organized into clusters, such that each cluster contained identical or fully contained transcripts, and thus each cluster represented a unique transcript. A transcript is considered supported by all three replicates if the corresponding cluster contained transcripts assembled from all three replicates.
