# Supplementary material for: Transcriptome analysis of two isolates of the tomato pathogen Cladosporium fulvum, uncovers genome-wide patterns of alternative splicing during a host infection cycle
Source: PLoS Pathog. 2024 Dec 18;20(12):e1012791. doi: 10.1371/journal.ppat.1012791 (PMC11694984; doi:10.1371/journal.ppat.1012791)
Supplement: S4 Fig — (PDF) [file ppat.1012791.s007.pdf]

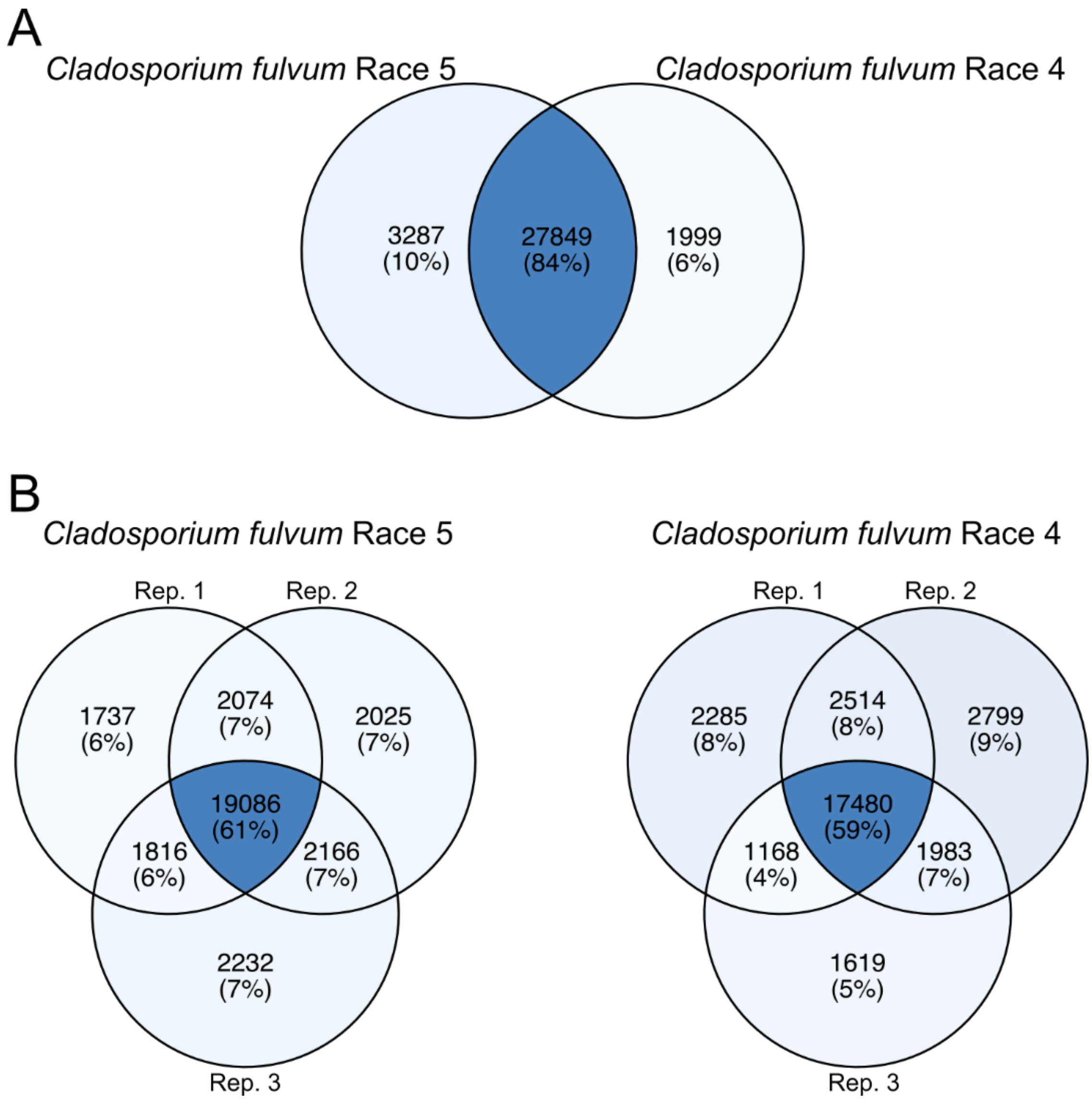

S4 Fig. The number of transcripts shared by *Cladosporium fulvum* isolates Race 5 and Race 4 increases when singleton transcripts were filtered out. (A) The total number of transcripts shared between isolates Race 5 and Race 4. The Venn diagram shows all uniquely assembled transcripts after combining all transcripts assembled across the seven sampled timepoints per infection and three infections (i.e. biological replicates), and subsequently removing singleton transcripts that were present in only one sample, i.e., present in only one replicate, in one timepoint, for one isolate. (B) The total number of assembled transcripts shared among biological replicates (Rep. 1, Rep. 2, and Rep. 3) for isolates Race 5 and Race 4. The Venn diagrams show all unique transcripts assembled for each replicate after combining all timepoints and removing transcripts that were present in only one sample. Darker colors of intersections indicate higher numbers.
