# Supplementary material for: Transcriptome analysis of two isolates of the tomato pathogen Cladosporium fulvum, uncovers genome-wide patterns of alternative splicing during a host infection cycle
Source: PLoS Pathog. 2024 Dec 18;20(12):e1012791. doi: 10.1371/journal.ppat.1012791 (PMC11694984; doi:10.1371/journal.ppat.1012791)
Supplement: S5 Fig — (PDF) [file ppat.1012791.s008.pdf]

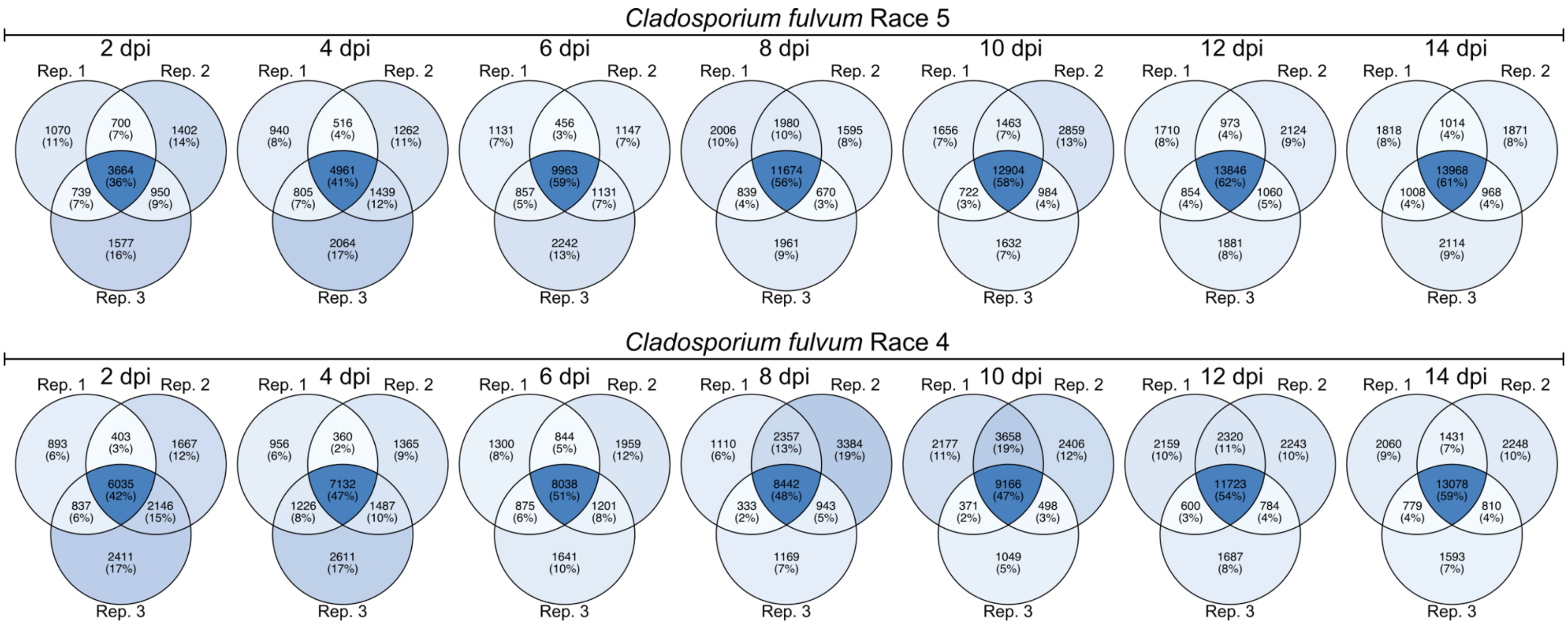

S5 Fig. The number of transcripts present in samples from all three tomato infections performed either with *Cladosporium fulvum* isolate Race 5 or Race 4, and in each of the seven infection timepoints that were sampled per infection, after filtering out singleton transcripts that were present in only one sample. Venn diagrams show the number of transcripts supported by one, two, or all three biological replicates (Rep. 1, Rep. 2, and Rep. 3) at each sampled infection timepoint (2, 4, 6, 8, 10, 12, 14 dpi) for isolates Race 5 and Race 4 after removing singleton transcripts that were present in only one sample, i.e., present in only one replicate, in one timepoint, for one isolate. Darker colors of intersections indicate higher numbers.
