# Supplementary material for: Transcriptome analysis of two isolates of the tomato pathogen Cladosporium fulvum, uncovers genome-wide patterns of alternative splicing during a host infection cycle
Source: PLoS Pathog. 2024 Dec 18;20(12):e1012791. doi: 10.1371/journal.ppat.1012791 (PMC11694984; doi:10.1371/journal.ppat.1012791)
Supplement: S6 Fig — (PDF) [file ppat.1012791.s009.pdf]

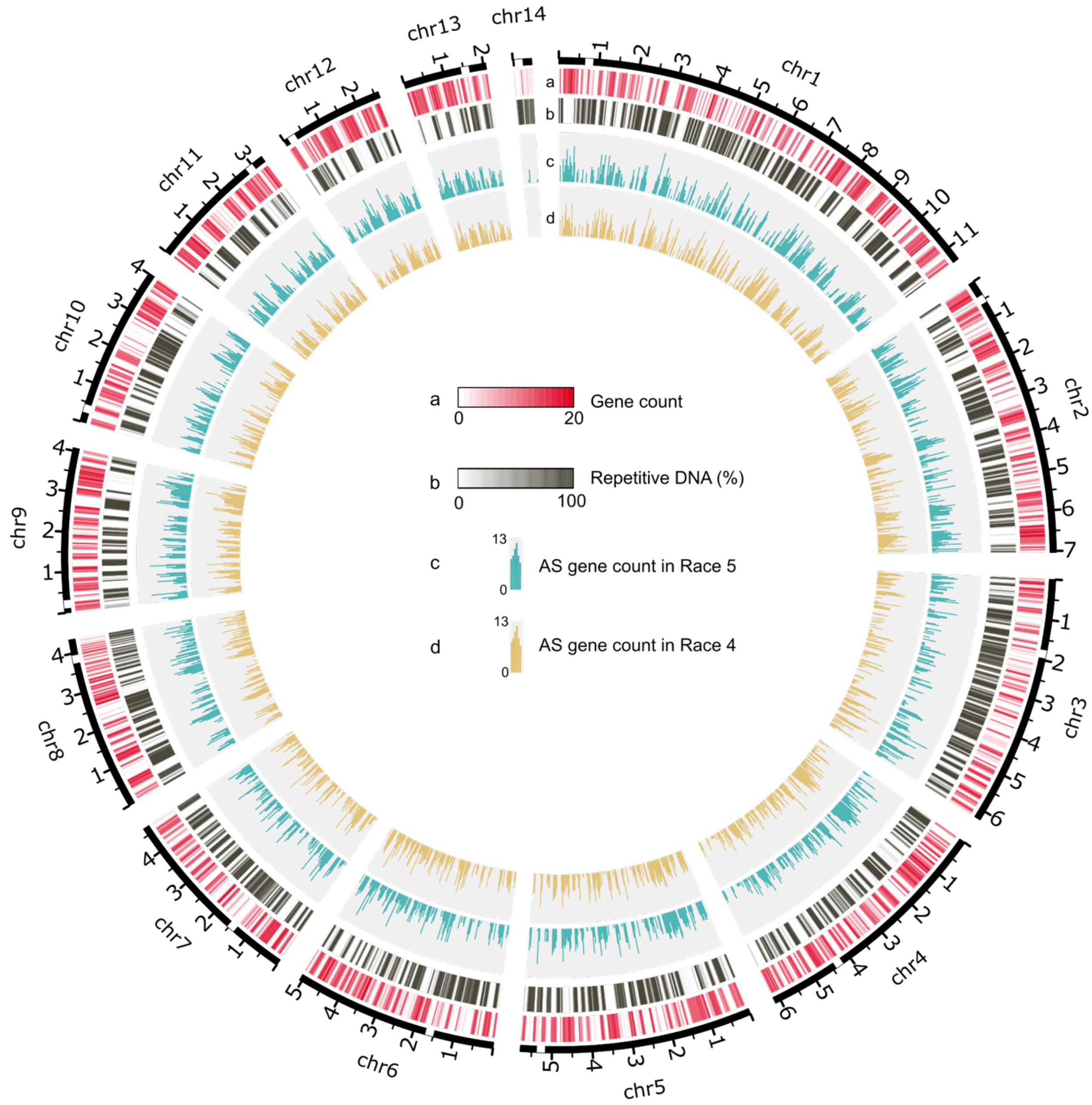

S6 Fig. The genomic distribution of genes predicted to be recurrently alternative spliced (AS) in *Cladosporium fulvum* isolates Race 5 and Race 4 during tomato infections. Circos plot showing the chromosomes of the reference genome of isolate Race 5. The two outermost tracks show the gene and repetitive DNA content, respectively. The predicted locations of centromeres on the chromosomes are indicated with white rectangles on the outermost axis. The histograms in the two innermost tracks represent the number of AS genes (0 to 13) from isolates Race 5 (in blue) and Race 4 (in yellow). Numbers in all tracks were calculated using a sliding window of 30 kb.
