# Supplementary material for: Transcriptome analysis of two isolates of the tomato pathogen Cladosporium fulvum, uncovers genome-wide patterns of alternative splicing during a host infection cycle
Source: PLoS Pathog. 2024 Dec 18;20(12):e1012791. doi: 10.1371/journal.ppat.1012791 (PMC11694984; doi:10.1371/journal.ppat.1012791)
Supplement: S7 Fig — (PDF) [file ppat.1012791.s010.pdf]

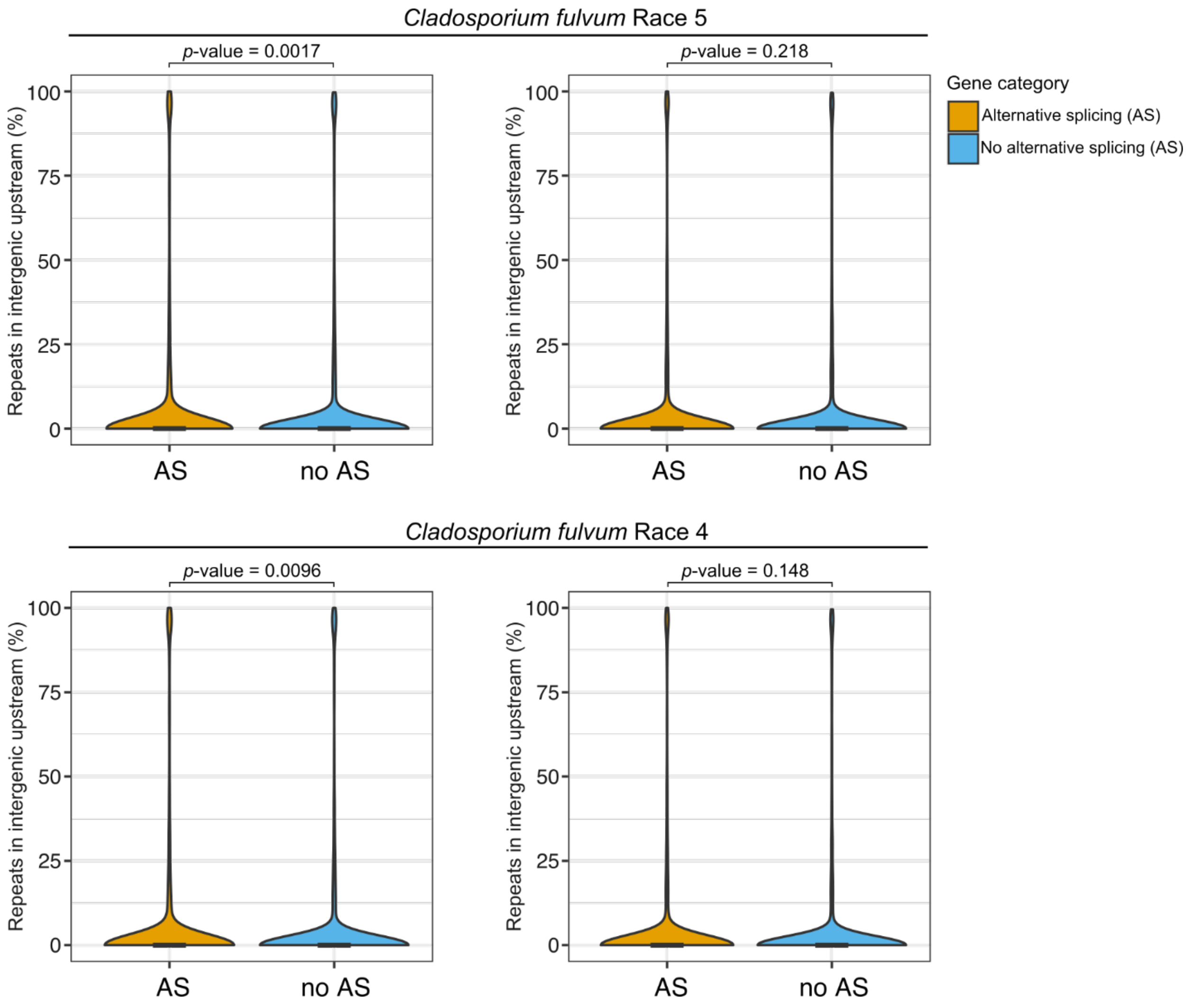

S7 Fig. The upstream genomic regions of alternative spliced (AS) genes in *Cladosporium fulvum* isolates Race 5 and Race 4, have higher amounts of repetitive DNA compared to the upstream genomic regions of non-AS genes. The violin plots show the distribution of the amount of repetitive DNA (i.e. predicted transposable elements) present in the up- and down-stream intergenic regions of genes with and without evidence of AS in isolates Race 5 and Race 4. The figure shows that the repetitive DNA content of upstream intergenic regions is significantly higher for AS genes (average= 6.26%) compared to non-AS genes (average= 5.5%) in isolate Race 5. Similar observations are made for the upstream intergenic regions of AS (average= 6.13%) and non-AS (average= 5.59%) genes of isolate Race 4. In contrast, no significant differences in the amount of repetitive DNA is seen between the downstream intergenic regions of AS genes (Race 5 average= 5.19%; Race 4 average = 5.23%) and non-AS genes (Race 5 average = 4.96%; Race 4 average = 4.94%). The p-values were obtained with the Wilcoxon rank sum test.
