# Supplementary material for: Transcriptome analysis of two isolates of the tomato pathogen Cladosporium fulvum, uncovers genome-wide patterns of alternative splicing during a host infection cycle
Source: PLoS Pathog. 2024 Dec 18;20(12):e1012791. doi: 10.1371/journal.ppat.1012791 (PMC11694984; doi:10.1371/journal.ppat.1012791)
Supplement: S8 Fig — (PDF) [file ppat.1012791.s011.pdf]

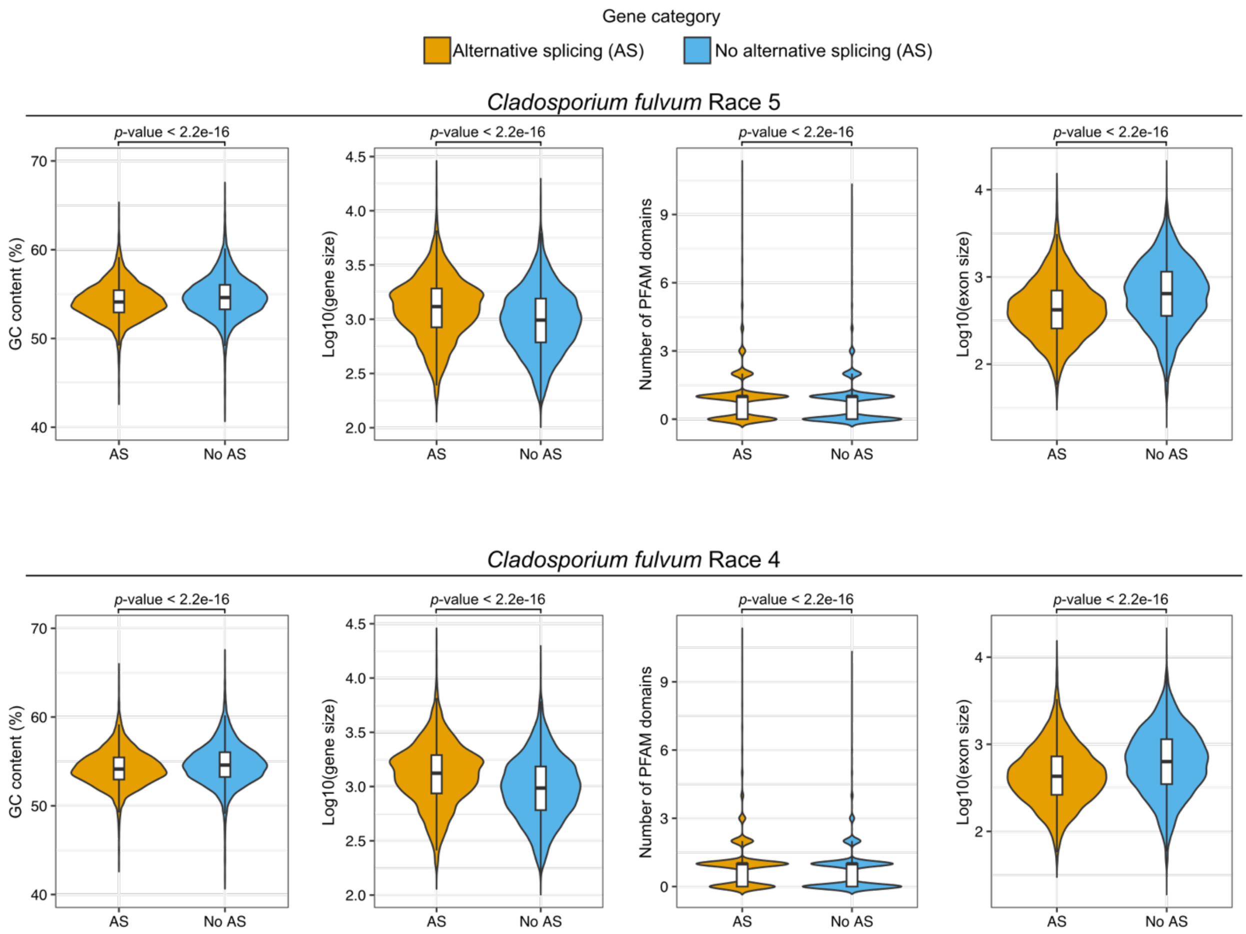

S8 Fig. Genes that are alternatively spliced (AS) in *Cladosporium fulvum* isolates Race 5 and Race 4 during tomato infections exhibit distinct physical characteristics as compared to non-AS genes. Violin plots showing the distributions of size, GC content, number of PFAM domains, and size of exons of AS genes as compared to non-AS genes. The entire gene sequences, including both exons and introns, were considered to calculate size and GC content, while only the coding sequences were used to determine the number of PFAM domains and size of exons. P-values were obtained using the Wilcoxon rank sum tests. Mean and median values of the distributions are shown in Table S10.
