# Supplementary material for: Transcriptome analysis of two isolates of the tomato pathogen Cladosporium fulvum, uncovers genome-wide patterns of alternative splicing during a host infection cycle
Source: PLoS Pathog. 2024 Dec 18;20(12):e1012791. doi: 10.1371/journal.ppat.1012791 (PMC11694984; doi:10.1371/journal.ppat.1012791)
Supplement: S10 Fig — (PDF) [file ppat.1012791.s013.pdf]

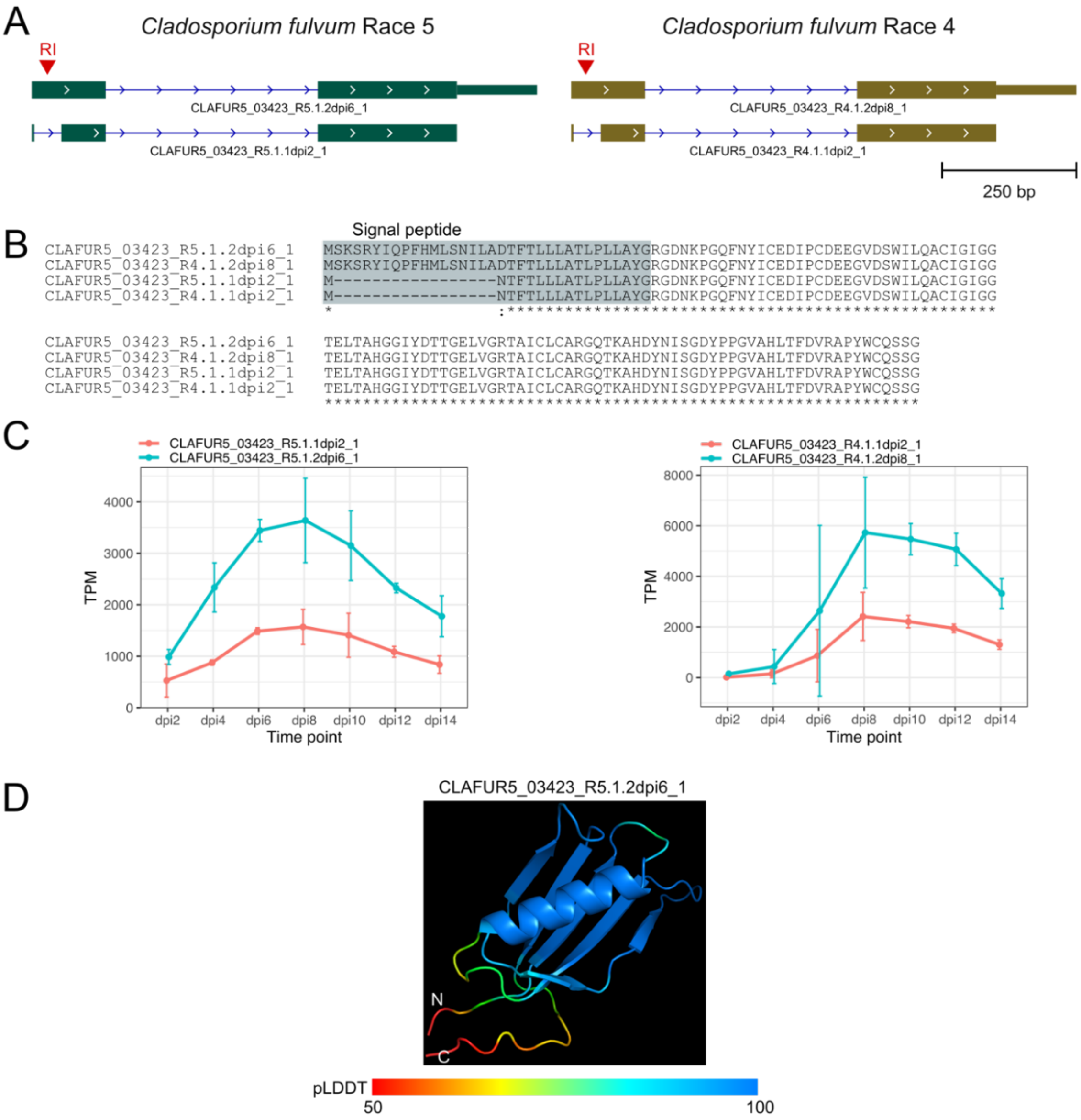

S10 Fig. Alternative splicing (AS) in the effector gene *Ecp5* of *Cladosporium fulvum* isolates Race 5 and Race 4. (A) An intro retention (IR) event results in the modification of the 17 amino acids after the intron splice site. (B) Multiple sequence alignment of the encoded protein isoforms of *Ecp5* from *C. fulvum* isolates Race 5 and Race 4. (C) Expression of *Ecp5* isoforms. In the line graphs, points represent the expression values in TPM (transcripts per million) of the individual transcripts across different timepoints of the infection. The standard deviation in the TPM values from three infections (i.e. biological replicates) is shown as vertical lines. The trends of transcript expression across time are shown as thick lines connecting the average TPM values for each individual transcript. (D) Structure of the mature protein sequence encoded by one of the *Ecp5* isoforms from *C. fulvum* isolate Race 5 predicted uisng AlphaFold2. As the RI event is predicted to affect only the signal peptide sequence, no changes in the mature protein sequences are expected. Protein structure was colored according to the predicted Local Distance Difference Test (pLDDT) values reported by AlphaFold2. i.e., dark blue indicates very high confidence (pLDDT > 90), light blue indicates high confidence (90 > pLDDT > 70), yellow indicates low confidence (70 > pLDDT > 50), and red indicates very low confidence (pLDDT < 50).
