# Supplementary material for: Transcriptome analysis of two isolates of the tomato pathogen Cladosporium fulvum, uncovers genome-wide patterns of alternative splicing during a host infection cycle
Source: PLoS Pathog. 2024 Dec 18;20(12):e1012791. doi: 10.1371/journal.ppat.1012791 (PMC11694984; doi:10.1371/journal.ppat.1012791)
Supplement: S11 Fig — (PDF) [file ppat.1012791.s014.pdf]

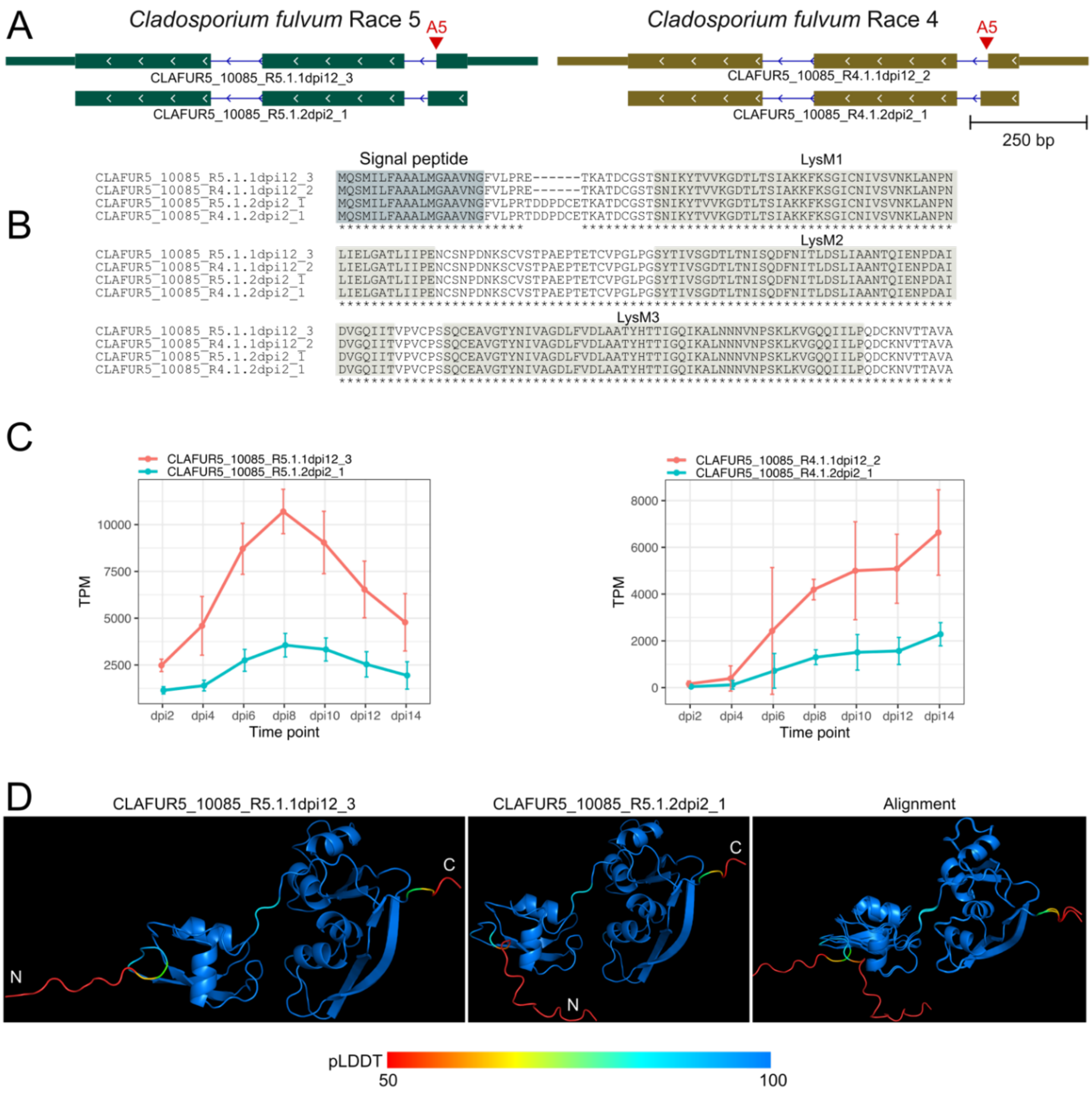

S11 Fig. Alternative splicing (AS) in the effector gene *Ecp6* of *Cladosporium fulvum* isolates Race 5 and Race 4. (A) An alternative 5' splice site (A5) event results in the modification of 6 amino acids after the splice site. (B) Multiple sequence alignment of the encoded protein isoforms of *Ecp6* from *C. fulvum* isolates Race 5 and Race 4. (C) Expression of *Ecp6* isoforms. In the line graphs, points represent the expression values in TPM (transcripts per million) of the individual transcripts across different timepoints of the infection. The standard deviation in the TPM values from three infections (i.e. biological replicates) is shown as vertical lines. The trends of transcript expression across time are shown as thick lines connecting the average TPM values for each individual transcript. (D) Structures of the mature protein sequences encoded by the *Ecp6* isoforms from *C. fulvum* isolate Race 5 predicted using AlphaFold2. The rightmost panel shows both protein structures aligned. The predicted structures for *Ecp6* isoforms from *C. fulvum* isolate Race 4 were identical to those from isolate Race 5, and thus were omitted. Protein structures were colored based on the predicted Local Distance Difference Test (pLDDT) values reported by AlphaFold2. i.e., dark blue indicates very high confidence (pLDDT > 90), light blue indicates high confidence (90 > pLDDT > 70), yellow indicates low confidence (70 > pLDDT > 50), and red indicates very low confidence (pLDDT < 50).
