# Supplementary material for: Transcriptome analysis of two isolates of the tomato pathogen Cladosporium fulvum, uncovers genome-wide patterns of alternative splicing during a host infection cycle
Source: PLoS Pathog. 2024 Dec 18;20(12):e1012791. doi: 10.1371/journal.ppat.1012791 (PMC11694984; doi:10.1371/journal.ppat.1012791)
Supplement: S13 Fig — (PDF) [file ppat.1012791.s016.pdf]

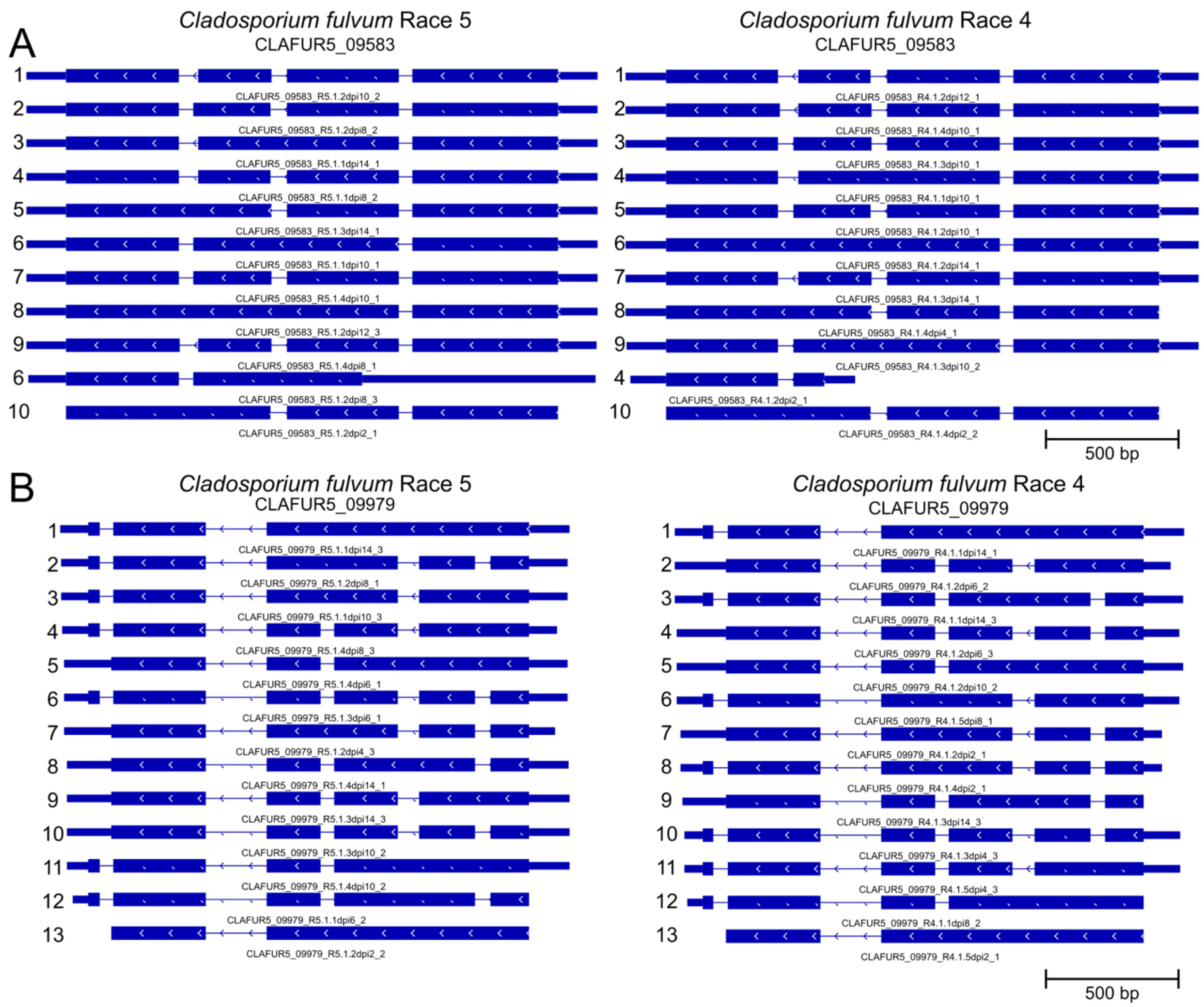

S13 Fig. Two genes encoding putative transcription factors in *Cladosporium fulvum* isolates Race 5 and Race 4, and their predicted protein isoforms produced via alternative splicing (AS) events. (A) AS in gene *CLAFUR5_09583* leads to 10 different protein isoforms, in both isolates Race 5 and Race 4. (B) AS in gene *CLAFUR5_09979* leads to 13 different protein isoforms, in both isolates Race 5 and Race 4. The number on the left-hand side counts the number of distinct protein isoforms produced via AS by each gene in each isolate. Image is exported from IGV. Thick rectangles represent coding sequences, whereas thinner rectangles represent untranslated regions, and lines represent introns.
