# Supplementary material for: Transcriptome analysis of two isolates of the tomato pathogen Cladosporium fulvum, uncovers genome-wide patterns of alternative splicing during a host infection cycle
Source: PLoS Pathog. 2024 Dec 18;20(12):e1012791. doi: 10.1371/journal.ppat.1012791 (PMC11694984; doi:10.1371/journal.ppat.1012791)
Supplement: S14 Fig — (PDF) [file ppat.1012791.s017.pdf]

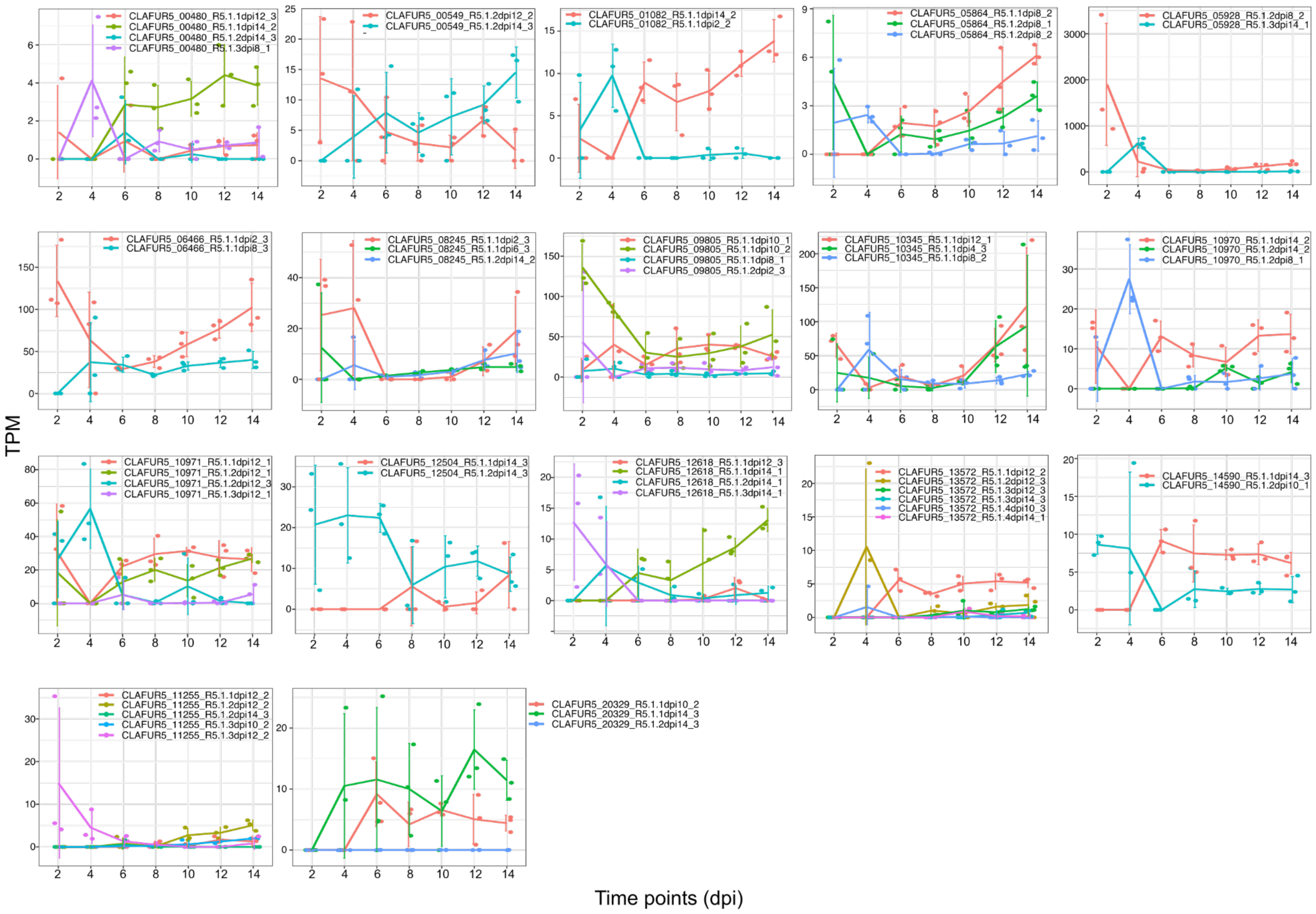
CLAFUR5_00480_R5.1.1dpi12_3
CLAFUR5_00480_R5.1.1dpi14_2
CLAFUR5_00480_R5.1.2dpi14_3
CLAFUR5_00480_R5.1.3dpi8_1
CLAFUR5_00549_R5.1.2dpi12_2
CLAFUR5_00549_R5.1.2dpi14_3
CLAFUR5_01082_R5.1.1dpi14_2
CLAFUR5_01082_R5.1.1dpi2_2
CLAFUR5_05864_R5.1.1dpi8_2
CLAFUR5_05864_R5.1.2dpi8_1
CLAFUR5_05864_R5.1.2dpi8_2
CLAFUR5_05928_R5.1.2dpi8_2
CLAFUR5_05928_R5.1.3dpi14_1
CLAFUR5_06466_R5.1.1dpi2_3
CLAFUR5_06466_R5.1.1dpi8_3
CLAFUR5_08245_R5.1.1dpi2_3
CLAFUR5_08245_R5.1.1dpi6_3
CLAFUR5_08245_R5.1.2dpi14_2
CLAFUR5_09805_R5.1.1dpi10_1
CLAFUR5_09805_R5.1.1dpi10_2
CLAFUR5_09805_R5.1.1dpi8_1
CLAFUR5_09805_R5.1.2dpi2_3
CLAFUR5_10345_R5.1.1dpi12_1
CLAFUR5_10345_R5.1.1dpi4_3
CLAFUR5_10345_R5.1.1dpi8_2
CLAFUR5_10970_R5.1.1dpi14_2
CLAFUR5_10970_R5.1.2dpi14_2
CLAFUR5_10970_R5.1.2dpi8_1
CLAFUR5_10971_R5.1.1dpi12_1
CLAFUR5_10971_R5.1.2dpi12_1
CLAFUR5_10971_R5.1.2dpi12_3
CLAFUR5_10971_R5.1.3dpi12_1
CLAFUR5_12504_R5.1.1dpi14_3
CLAFUR5_12504_R5.1.2dpi14_3
CLAFUR5_12618_R5.1.1dpi12_3
CLAFUR5_12618_R5.1.1dpi14_1
CLAFUR5_12618_R5.1.2dpi14_1
CLAFUR5_12618_R5.1.3dpi14_1
CLAFUR5_13572_R5.1.1dpi12_2
CLAFUR5_13572_R5.1.2dpi12_3
CLAFUR5_13572_R5.1.3dpi12_3
CLAFUR5_13572_R5.1.3dpi14_3
CLAFUR5_13572_R5.1.4dpi10_3
CLAFUR5_13572_R5.1.4dpi14_1
CLAFUR5_14590_R5.1.1dpi14_3
CLAFUR5_14590_R5.1.2dpi10_1
CLAFUR5_11255_R5.1.1dpi12_2
CLAFUR5_11255_R5.1.2dpi12_2
CLAFUR5_11255_R5.1.2dpi14_3
CLAFUR5_11255_R5.1.3dpi10_2
CLAFUR5_11255_R5.1.3dpi12_2
CLAFUR5_20329_R5.1.1dpi10_2
CLAFUR5_20329_R5.1.1dpi14_3
CLAFUR5_20329_R5.1.2dpi14_3
TPM
Time points (dpi)

S14. Fig. Genes from *Cladosporium fulvum* isolate Race 5 with significant evidence of differential isoform usage at the transcript level during disease progression, which are common to both isolates. The line graphs show 17 AS genes from isolate Race 5 that produce transcripts whose relative abundance significantly changes during the infection. In the line graphs, the points represent the expression values in TPM (transcripts per million) of the individual transcripts across different timepoints of the infection. Standard deviation in the TPM values from three infections (i.e. biological replicates) is shown as vertical lines. The trends of transcript expression across time are shown as thick lines connecting the average TPM values for each individual transcript.
