# Supplementary material for: Transcriptome analysis of two isolates of the tomato pathogen Cladosporium fulvum, uncovers genome-wide patterns of alternative splicing during a host infection cycle
Source: PLoS Pathog. 2024 Dec 18;20(12):e1012791. doi: 10.1371/journal.ppat.1012791 (PMC11694984; doi:10.1371/journal.ppat.1012791)
Supplement: S15 Fig — (PDF) [file ppat.1012791.s018.pdf]

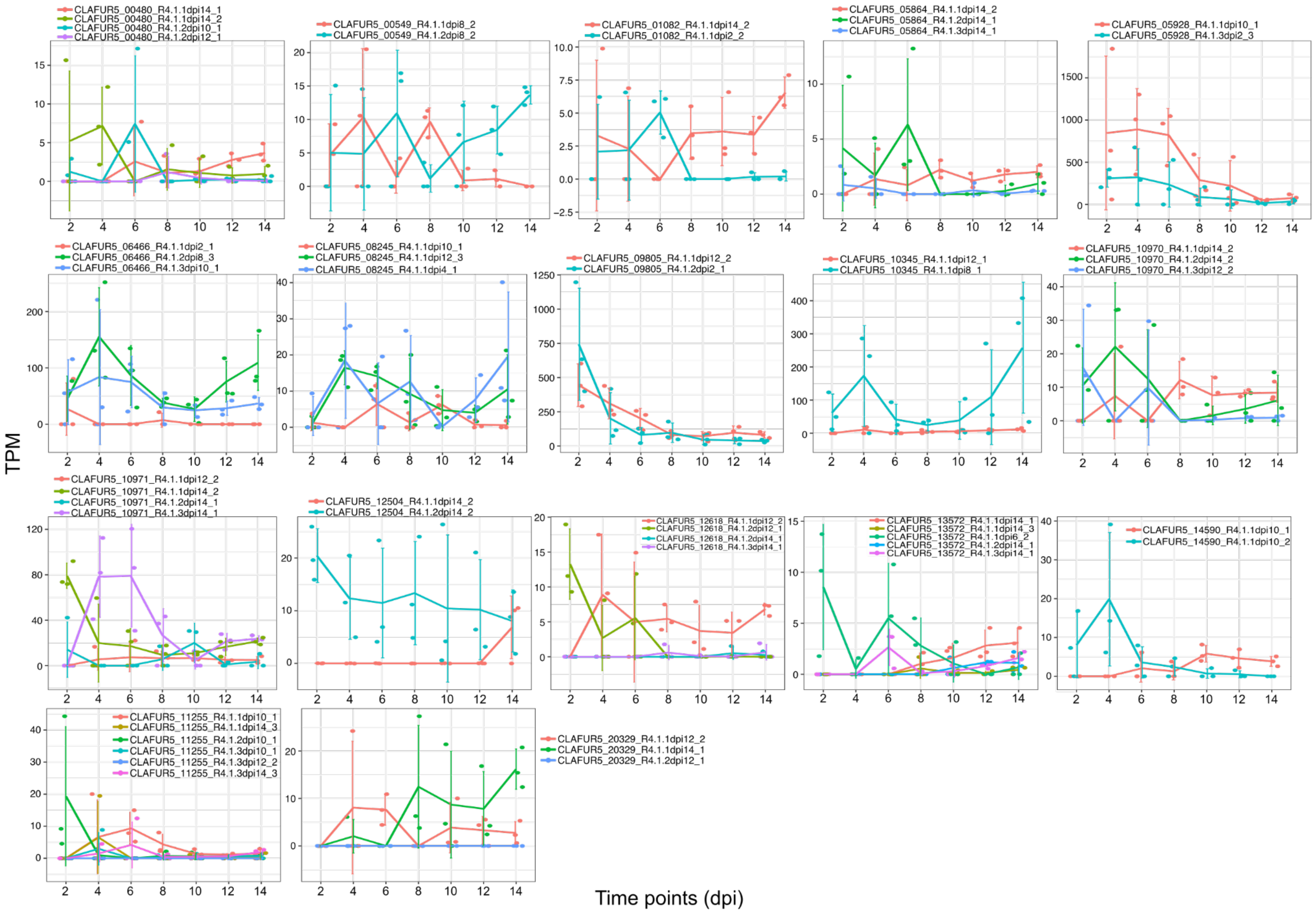
CLAFUR5_00480_R4.1.1dpi14_1
CLAFUR5_00480_R4.1.1dpi14_2
CLAFUR5_00480_R4.1.2dpi10_1
CLAFUR5_00480_R4.1.2dpi12_1
CLAFUR5_00549_R4.1.1dpi8_2
CLAFUR5_00549_R4.1.2dpi8_2
CLAFUR5_01082_R4.1.1dpi14_2
CLAFUR5_01082_R4.1.1dpi2_2
CLAFUR5_05864_R4.1.1dpi14_2
CLAFUR5_05864_R4.1.2dpi14_1
CLAFUR5_05864_R4.1.3dpi14_1
CLAFUR5_05928_R4.1.1dpi10_1
CLAFUR5_05928_R4.1.3dpi2_3
CLAFUR5_06466_R4.1.1dpi2_1
CLAFUR5_06466_R4.1.2dpi8_3
CLAFUR5_06466_R4.1.3dpi10_1
CLAFUR5_08245_R4.1.1dpi10_1
CLAFUR5_08245_R4.1.1dpi12_3
CLAFUR5_08245_R4.1.1dpi4_1
CLAFUR5_09805_R4.1.1dpi12_2
CLAFUR5_09805_R4.1.2dpi2_1
CLAFUR5_10345_R4.1.1dpi12_1
CLAFUR5_10345_R4.1.1dpi8_1
CLAFUR5_10970_R4.1.1dpi14_2
CLAFUR5_10970_R4.1.2dpi14_2
CLAFUR5_10970_R4.1.3dpi12_2
TPM
CLAFUR5_10971_R4.1.1dpi12_2
CLAFUR5_10971_R4.1.1dpi14_2
CLAFUR5_10971_R4.1.2dpi14_1
CLAFUR5_10971_R4.1.3dpi14_1
CLAFUR5_12504_R4.1.1dpi14_2
CLAFUR5_12504_R4.1.2dpi14_2
CLAFUR5_12618_R4.1.1dpi12_2
CLAFUR5_12618_R4.1.2dpi12_1
CLAFUR5_12618_R4.1.2dpi14_1
CLAFUR5_12618_R4.1.3dpi14_1
CLAFUR5_13572_R4.1.1dpi14_1
CLAFUR5_13572_R4.1.1dpi14_3
CLAFUR5_13572_R4.1.1dpi6_2
CLAFUR5_13572_R4.1.2dpi14_1
CLAFUR5_13572_R4.1.3dpi14_1
CLAFUR5_14590_R4.1.1dpi10_1
CLAFUR5_14590_R4.1.1dpi10_2
CLAFUR5_11255_R4.1.1dpi10_1
CLAFUR5_11255_R4.1.1dpi14_3
CLAFUR5_11255_R4.1.2dpi10_1
CLAFUR5_11255_R4.1.3dpi10_1
CLAFUR5_11255_R4.1.3dpi12_2
CLAFUR5_11255_R4.1.3dpi14_3
CLAFUR5_20329_R4.1.1dpi12_2
CLAFUR5_20329_R4.1.1dpi14_1
CLAFUR5_20329_R4.1.2dpi12_1
Time points (dpi)

S15 Fig. Genes from *Cladosporium fulvum* isolate Race 4 with significant evidence of differential isoform usage at the transcript level during disease progression, which are common to both isolates. The line graphs show 17 AS genes from isolate Race 4 that produce transcripts whose relative abundance significantly changes during the infection. In the line graphs, the points represent the expression values in TPM (transcripts per million) of the individual transcripts across different timepoints of the infection. Standard deviation in the TPM values from three infections (i.e. biological replicates) is shown as vertical lines. The trends of transcript expression across time are shown.
