# Supplementary material for: Transcriptome analysis of two isolates of the tomato pathogen Cladosporium fulvum, uncovers genome-wide patterns of alternative splicing during a host infection cycle
Source: PLoS Pathog. 2024 Dec 18;20(12):e1012791. doi: 10.1371/journal.ppat.1012791 (PMC11694984; doi:10.1371/journal.ppat.1012791)
Supplement: S16 Fig — (PDF) [file ppat.1012791.s019.pdf]

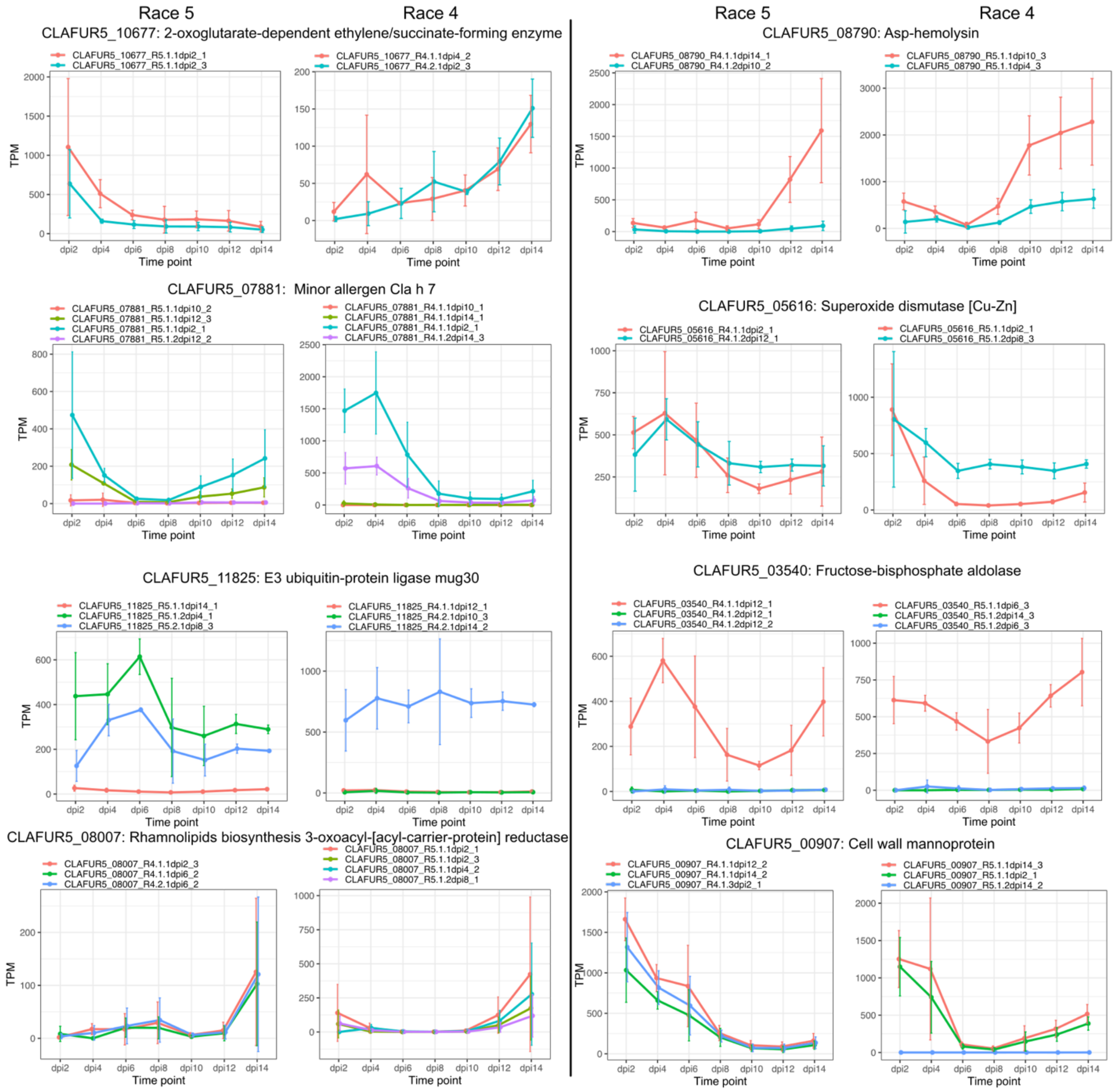

S16 Fig. Examples of genes from *Cladosporium fulvum* producing isoforms with unsual expression patterns during the infection process. Pints in the line graphs represent the expression values in TPM (transcripts per million) of the individual transcripts at various timepoints of the infection. The standard deviation in the TPM values from three infections (i.e. biological replicates) is shown as vertical lines. The trends of transcript expression across time are shown and the predicted functions or the enzymes that are by the genes are noted in each plot.
