# Supplementary material for: Transcriptome analysis of two isolates of the tomato pathogen Cladosporium fulvum, uncovers genome-wide patterns of alternative splicing during a host infection cycle
Source: PLoS Pathog. 2024 Dec 18;20(12):e1012791. doi: 10.1371/journal.ppat.1012791 (PMC11694984; doi:10.1371/journal.ppat.1012791)
Supplement: S17 Fig — (PDF) [file ppat.1012791.s020.pdf]

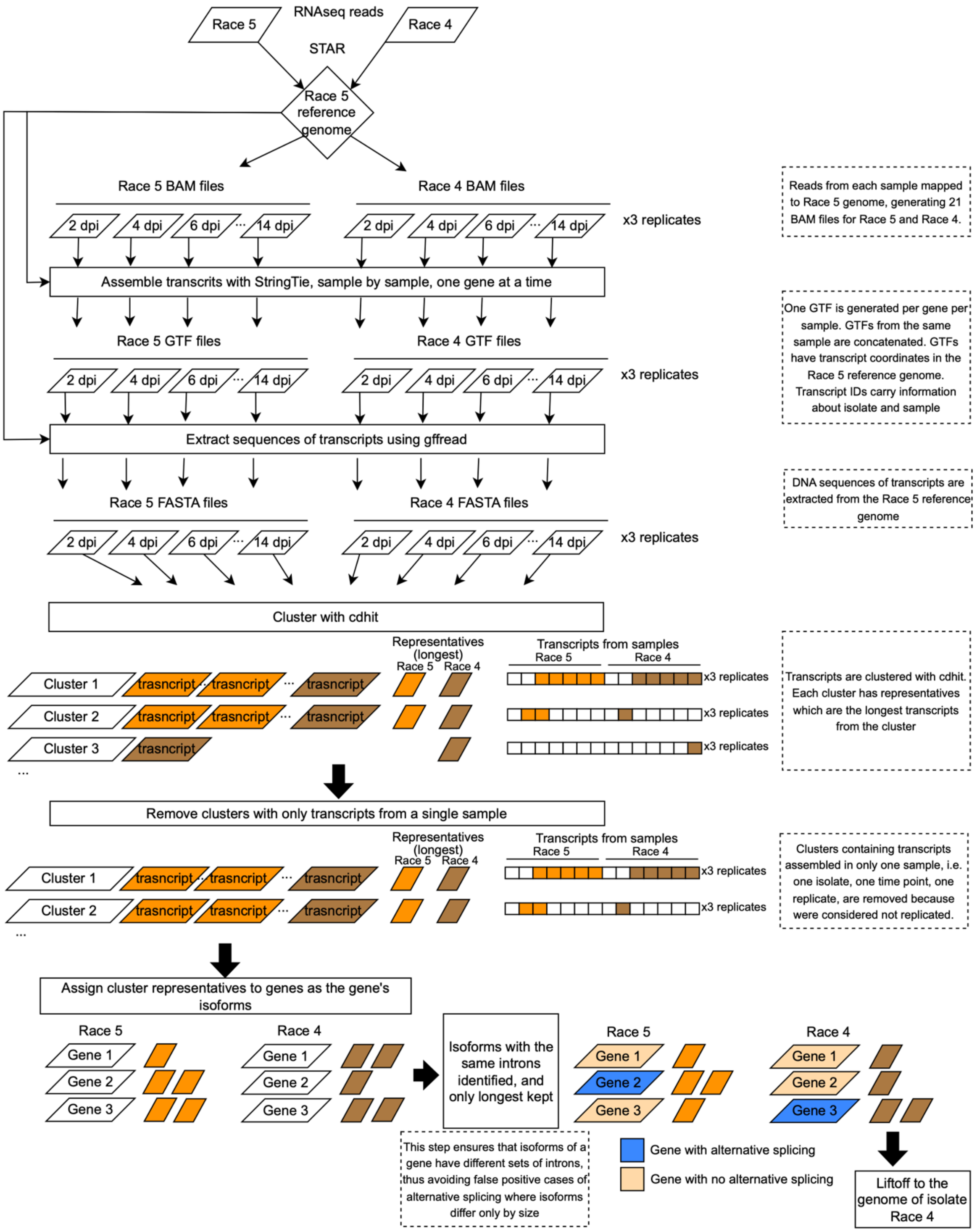

S17 Fig. Flowchart summarizing the steps performed to assemble transcripts of *Cladosporium fulvum* isolates Race 5 and Race 4, and identify genes predicted to undergo alternative splicing. Reads were mapped to the genome of isolate Race 5 and transcripts were reconstructed. To minimize the occurrence of chimeric transcripts from genes that are physically close to each other, transcripts were reconstructed one gene at a time. The resulting transcripts were concatenated into single GTF files, resulting in 42 GTF

files (one per sample) with assembled transcripts. Using the genome of Race 5 isolate as a reference, the sequences of the transcripts were extracted into FASTA files, and then clustered with cdhit. Each cluster included transcripts from Race 5 and/or Race 4, assembled from one or more time points or replicates. Clusters were represented by the longest transcripts in them and clusters with transcripts assembled based on reads of a single sample were removed. The representative transcripts in each cluster were then assigned to their genes of origin. A final filtering step was incorporated to identify transcripts from the same gene that had the same set of introns (same number and coordinates). Only the longest of such transcripts were kept, thereby safeguarding that transcript isoforms of a gene have different sets of introns, which prevents false positive cases of alternative splicing in which transcripts differ only by their size.
