# Supplementary material for: Transcriptome analysis of two isolates of the tomato pathogen Cladosporium fulvum, uncovers genome-wide patterns of alternative splicing during a host infection cycle
Source: PLoS Pathog. 2024 Dec 18;20(12):e1012791. doi: 10.1371/journal.ppat.1012791 (PMC11694984; doi:10.1371/journal.ppat.1012791)
Supplement: Attachment — Submitted filename: Responce to Reviewer Comments.pdf [file ppat.1012791.s039.pdf]

Response to Reviewer Comments

Reviewer's Responses to Questions

Part I - Summary

Please use this section to discuss strengths/weaknesses of study, novelty/significance, general execution and scholarship.

Reviewer #1: alternative splicing is an aspect of fungal pathogenomics that has not been well-studied so far... and this study is an interesting contribution to the knowledge of this topic

the manuscript was well written and of good quality overall

Response: We sincerely appreciate the reviewer's time, effort, and feedback on our manuscript. We are also grateful that the reviewer recognizes that our study makes a significant contribution to the field of fungal pathogenomics and alternative splicing (AS). In the revised manuscript, we have carefully considered and addressed each comment and suggestion made by the reviewer, and we believe that the revisions that were made to the manuscript have improved the quality and clarity of our work. We hope that the reviewer finds our revisions and responses satisfactory as well.

Reviewer #2: The manuscript by Zaccaron et al examines alternative splicing during a host infection cycle for two different isolates of the tomato pathogen Cladosporium fulvum. The authors have generated a highly interesting dataset that will be of interest not just for the study of alternative splicing but also for examining transcriptome dynamics in the species. The analyses are all expertly done and the manuscript very clearly written.

Response: We are thankful to the reviewer for their thorough review of our manuscript and for acknowledging the quality of our analyses and the clarity of our work. In the revised manuscript, we have made efforts to carefully consider and address all the reviewer's comments and suggestions. We hope that the reviewer finds our responses to their concerns reasonable, and the revisions to our manuscript acceptable.

Reviewer #3: The study examines alternative splicing (AS) in the two fungal isolates, Race 4 and 5, of Cladosporium fulvum at seven timepoints of infection in tomato. The results revealed a relatively high percentage of AS genes in this fungus and overall similar patterns of AS in the two isolates. Interestingly, the study found that AS genes were enriched in repeat-rich chromosomes and had longer upstream intergenic regions. The exception was found in a dispensable chromosome. Differential isoform usage (DIU) for all pair-wise comparisons were performed and isoform switching events were identified.
AS analysis is not a straightforward process. The approaches used well reasonably selected.

Response: We are grateful to the reviewer for meticulously evaluating our manuscript, and we appreciate their recognition of the complexity of the analysis in alternative splicing (AS), which highlights the challenges of our work. In response to the reviewer's comments and suggestions, we have made substantial revisions to the manuscript, aiming to address the points raised by the reviewer and further clarify our methods and findings. We believe that the revisions have significantly improved the manuscript's quality, and we sincerely hope that the reviewer finds our revisions and responses both reasonable and satisfactory.

Part II – Major Issues: Key Experiments Required for Acceptance

Please use this section to detail the key new experiments or modifications of existing experiments that should be absolutely required to validate study conclusions. Generally, there should be no more than 3 such required experiments or major modifications for a "Major Revision" recommendation. If more than 3 experiments are necessary to validate the study conclusions, then you are encouraged to recommend "Reject".

Reviewer #1: while the biological question of this study is intron splice variation… my main question is whether a potential technical issue with the use of cdhit may have influenced the interpretation of AS transcript heterogeneity. that is, whether post transcriptional mRNA sequence modifications that occur naturally, or perhaps triggered by infection, might be falsely counted as alternate transcripts based on altered sequence, but due to SNP changes rather than exon splicing/structural (indel?) changes. authors please comment on the potential for this based on their methods, and if it is a potential issue, address it accordingly by distinguishing SNP-differentiated from intron-differentiated alternate transcripts

Response: We thank the reviewer for expressing their concern. The reviewer's point about polymorphisms between the Race 4 and Race 5 isolates is indeed relevant, as it could impact the results of the clustering with CD-HIT. To address this challenge, we used the genome of Race 5 as a reference to assemble the transcripts of both isolates, which allowed us to obtain their transcripts in GTF format based on a single coordinate system. The transcript sequences were then extracted from the reference genome of Race 5, enabling us to cluster the transcripts based on their sequences using CD-HIT, without the polymorphisms between the two isolates affecting the clustering results. Next, transcripts from Race 4 were mapped to the Race 4 genome using Liftoff, and their sequences were extracted from this genome. Finally, ORFs were predicted in the transcripts. We trust that this approach enabled us to minimize potential issues with polymorphisms between Race 5 and Race 4 affecting the transcript heterogeneity between the two isolates. We also acknowledge that some steps in our methodology may be less clear or non-intuitive. Therefore, to facilitate comprehension, we have included a new supplementary figure, S17 Fig, in the revised manuscript, which illustrates a flowchart of the steps performed.

I would have liked to see more detail (and a figure) describing how AS occurs in known (not candidates) effectors of Cfulvum, as I understand the results there are only a few affected by AS, but as several effectors in Cfulvum are well studied this should be quite interesting
i.e. what is the sequence pre/post AS, which version occurs during infection or non-infection, how functional motifs and known/predicted 3D structure is altered by inclusion/exclusion of the intron regions, etc…

Response: We thank the reviewer for this valuable suggestion. We agree that providing more details about the potential impact of AS on known effector genes of *C. fulvum* would add some extra value to our study and offer new insights into AS in effector genes, which is an understudied area. Upon further inspection of our data, we observed that AS affected the mature proteins of Ecp1, Ecp6, and Ecp12, as well as the signal peptide of Ecp5. Specifically, expression analysis indicated that the longest isoform of Ecp5, carrying 17 extra amino acids in the signal peptide, was preferentially expressed. For Ecp6, the shortest isoform which is missing six amino acids between the signal peptide and the first LysM conserved domain, was the one preferentially expressed. In contrast, no isoform from Ecp1 and Ecp12 was clearly preferentially expressed in planta. These findings are described in lines 242-249 of the revised manuscript. Additionally, we have added new supplementary figures showing the exon/intron structure of the isoforms, their aligned amino acid sequences, the expression of the isoforms during infection, and their 3D structures predicted with AlphaFold2. These are presented for Ecp1 (S9 Fig), Ecp5 (S10 Fig), Ecp6 (S11 Fig), and Ecp12 (S12 Fig).

Reviewer #2: The authors have done a great job examining the surprising levels of alternative splicing in relation to the functions of genes and their locations in the genome. My only major comment is that it would have been interesting to also see a figure where the frequency of alternative splicing is also examined in light of the physical characteristics of genes (e.g., their length, number of domains, GC content, etc.). Given that some of the events seem to be associated with transcriptional noise / heterogeneity, it may very well be that this noise is in turn associated with certain gene characteristics.

Response: We thank the reviewer for the suggestion and for recognizing the large amount of effort placed in analyzing genes undergoing alternative splicing (AS) and their features. We agree that adding more details in our manuscript about the frequency of AS and physical characteristics of AS genes will offer further insights into its occurrence. Therefore, in the revised manuscript, we report a comparison of GC content, size, number of conserved PFAM domains, and size of exons between genes with and without evidence of AS. The analysis revealed that AS genes had significantly lower GC content, were longer, contained more conserved PFAM domains, and had shorter exons compared to non-AS genes. These results are reported in the revised manuscript in lines 209-215. Additionally, we have included a new supplementary figure, S8 Fig., illustrating these comparisons, as well as a new supplementary table, S10 Table, presenting the mean and median values of the distributions. We hope that these new data further illuminate the features of AS genes in the pathogen during infections.

Reviewer #3: Here is my major concerns.

1. The study relies on the quality of transcriptomic assemblies. Although the package StringTie is considered as a good reference-based transcriptomic assembler and the authors employed a gene-by-gene assembly strategy to reduce the artifact, the assembly is probably still very noisy.

Response: We thank the reviewer for their comment. We are aware that transcriptome assembly can be noisy and potentially inaccurate, but to address these concerns, we implemented several careful strategies in our analyses; First, we used the latest version of StringTie, a state-of-the-art assembler, and performed the assembly gene-by-gene, to prevent chimeric transcripts resulting from genes with overlapping UTRs. Second, we made efforts to minimize transcripts arising from noise. For instance, during clustering analysis, we removed transcripts assembled from reads of only one sample, ensuring reproducibility. Additionally, we implemented a filtering step that compared introns of isoforms within a gene, removing isoforms with identical introns (i.e. with the same number and start/end coordinates), except for the longest one. This filter minimizes noise from poorly predicted transcript start and end coordinates.

We acknowledge that AS is poorly studied in fungi, and to our knowledge, StringTie and other transcript assemblers have not been properly evaluated for genes from filamentous fungi. However, since fungal gene transcripts are likely easier to assemble compared to those of plants and animals, StringTie is expected to perform well with fungal transcripts as well. As shown in Fig 1, almost all *C. fulvum* genes have 3 or fewer introns. This relatively simple gene structure likely facilitates transcript assembly compared to plant and animal genes, which typically have more than 5 introns, thereby increasing the likelihood of AS occurring. Indeed, as reported in the revised manuscript (lines 250-253), approximately 70% of the AS genes in *C. fulvum* produced only two isoforms, and over 96% of the AS events identified in both Race 5 and Race 4 isolates corresponded to alternative 3'/5' splice sites or retained introns (Table S7). The complexity of transcripts resulting from the presence/absence of entire exons is essentially not detected in *C. fulvum*.

In summary, considering our efforts to remove noise-induced transcripts and the expectation that most AS genes in *C. fulvum* express relatively simple isoforms, we anticipate that StringTie performed adequately for our analyses and has not severely affected the results presented in the manuscript.

2. The study also did not consider the polymorphisms between the Race 4 and Race 5 genomes. Structural variation and other polymorphisms could significantly influence the results, which likely affects the conclusions drawn in the manuscript.

Response: Thank you for the comment. Polymorphisms in the genes of Race 4 and Race 5 were indeed a concern when developing our methodology to identify unique and shared transcripts between these isolates. We considered two approaches for comparing transcripts from Race 4 and Race 5, i.e. (i) based on their coordinates, and (ii) based on their nucleotide sequences. Each approach presented its own challenges. For instance, in the first approach, genomic coordinates could differ between isolates, and INDELs within introns could subsequently alter the coordinates of downstream exons. In the second approach, differences in nucleotide sequences of transcripts from orthologous genes in Race 4 and Race 5 could be due to polymorphisms (SNPs, INDELs) or differences in splicing. To address these challenges, we implemented the following strategy; First, we used the Race 5 genome as reference to assemble transcripts from both isolates, obtaining transcripts in GTF format based on a single coordinate system. Next, transcript sequences were extracted from the Race 5 reference genome, and transcripts were clustered based on their sequences using CD-HIT, thereby avoiding the impact of polymorphisms on the clustering results. The clustering results were then used to predict shared and unique transcripts between the two isolates. Race 4 transcripts were subsequently mapped to the Race 4 genome using Liftoff, and their sequences were extracted from this genome. Finally, ORFs were predicted in these transcripts. We believe that this approach effectively minimized potential issues with polymorphisms between Race 5 and Race 4 affecting the transcript heterogeneity between the two isolates.

As demonstrated in our previous study (Zaccaron and Stergiopoulos 2024), *C. fulvum* has a stable gene complement with less than 2% of accessory genes, while nearly all structural variations do not affect genes and are mostly located in transposon-rich regions. Moreover, as reported in the revised manuscript (lines 97-102), after mapping the annotated genes from Race 4 to the Race 5 genome, we observed that 99.9% of one-to-one orthologous gene pairs from Race 4 and Race 5 had the same number of introns with identical start and end coordinates. Thus, it is unlikely that major differences in content and gene structures would arise from structural variations between Race 4 and Race 5.

To summarize, we used Race 5 as a reference to identify which transcripts were the same or different within and between the isolates. However, ORFs and downstream analyses were performed after the Liftoff step, which extracted the transcript sequences from the Race 4 genome. We understand that some steps in our methodology may be less clear or non-intuitive. Therefore, to facilitate comprehension, we have included a new supplementary figure, S17 Fig, in the revised manuscript that illustrates a flowchart of our strategy.

References:

Zaccaron, A. Z., and Stergiopoulos, I. 2024. Analysis of five near-complete genome assemblies of the tomato pathogen *Cladosporium fulvum* uncovers additional accessory chromosomes and structural variations induced by transposable elements effecting the loss of avirulence genes. BMC Biol. 22:25

3. The manuscript could be improved if time-series expression patterns of AS isoforms could be described. It may identify genes showing interesting changing patterns over infection.

Response: We thank the reviewer for the suggestion. In the revised manuscript, we have included several new supplementary figures to illustrate the expression patterns of isoforms from various genes. These include (i) previously characterized effectors of *C. fulvum* undergoing AS, such as *Ecp1* (S9 Fig), *Ecp5* (S10 Fig), *Ecp6* (S11 Fig), and *Ecp12* (S12 Fig), and (ii) eight non-effector genes with distinct expression patterns (S16 Fig), two of which are particularly interesting due to their contrasting expression patterns between Race 5 and Race 4. These are gene CLAFUR5_10677, encoding a predicted 2-oxoglutarate-dependent ethylene/succinate-forming enzyme, whose expression decreases over time in Race 5 while increasing in Race 4, and gene CLAFUR5_03540, encoding a putative fructose-bisphosphate aldolase, which produces

three AS isoforms one of which shows an S-shaped expression pattern in Race 5 but a V-shaped expression pattern in Race 4. A brief description of these unusual expression patterns is included in the revised manuscript, in lines 314-321. We hope that the reviewer finds the new figures and analyses informative, as they provide additional insights into the complex regulation of gene expression and AS in *C. fulvum*.

Part III – Minor Issues: Editorial and Data Presentation Modifications

Please use this section for editorial suggestions as well as relatively minor modifications of existing data that would enhance clarity.

Reviewer #1: the figure images as uploaded to the combined pdf are very low resolution and need to be improved

Response: We thank the reviewer for bringing this issue to our attention. We apologize for the low resolution of the figures in the combined PDF, which undoubtedly made the review process more challenging. We have increased the resolution of the figures to 600 dpi and saved them as .tiff files. Figures were also assessed that they meet the technical requirements of PLOS Pathogens using the PACE software provided by the journal. Although all figures passed the assessment, we noticed that the resolution of some figures is still not optimal when the files are converted to .pdf during the submission process. To avoid this problem, we kindly ask the reviewer to use the download link located at the top right-hand side of each figure to download the original .tiff image to their computer.

Reviewer #2: Line 3: “higher eukaryotes” - there are no “higher” and “lower” organisms; better say “plants and mammals”, which I believe is what you mean.

Response: We thank the reviewer for this insightful comment. Indeed, our use of "higher eukaryotes" was intended to refer to plants and mammals. In the revised manuscript, we have replaced the term "higher eukaryotes" with the more specific terms such as "plants and animals" (Line 3). We have also thoroughly reviewed the entire manuscript to ensure that no other instances of "higher eukaryotes" remain, thus improving clarity and precision throughout the text.

Line 64: "adaptions" -> "adaptations"

Response: This typo was corrected in the revised manuscript (line 65).

Line 373: “in contract” -> “in contrast”

Response: This typo was corrected in the revised manuscript (line 393).

Reviewer #3: Other comments:

1. “The transcript isoforms were then mapped to the genes of each isolate, and genes associated with more than one transcript isoform were now essentially considered as AS.”

How about partial assembled transcripts? For example, if a transcript is shorter than the full transcript, would the gene be considered to be an AS gene?

Response: We thank the reviewer for this interesting question and the opportunity to clarify this point. During our analyses, we indeed encountered situations similar to what the reviewer describes, as some assembled transcripts were highly similar but differed in their start or end coordinates.

To address this issue, we implemented a filtering step based on the reasoning that start and end coordinates of transcripts are often difficult to predict accurately when assembling them from short reads, while intron coordinates are generally more reliable indicators of transcript structure. Our subsequent filtering process consisted of comparing the intron coordinates, or absence thereof, of the transcripts and included (i) grouping together transcripts with identical sets of introns (i.e. the same number of introns and same

start/end coordinates for all introns) that differed only in their start and end coordinates, and (ii) retaining only the longest transcript from each group. This filtering step also applies to transcripts without introns. For example, if multiple isoforms are assembled for an intronless gene, only the longest of them is kept, and the gene is not considered to undergo AS. We present a figure below to illustrate this filtering process, while a complete description of this methodology can be found in the methods section of the revised manuscript (lines 483-490). We believe that this approach minimizes artifacts from transcript assembly, while retaining biologically relevant information about AS events.

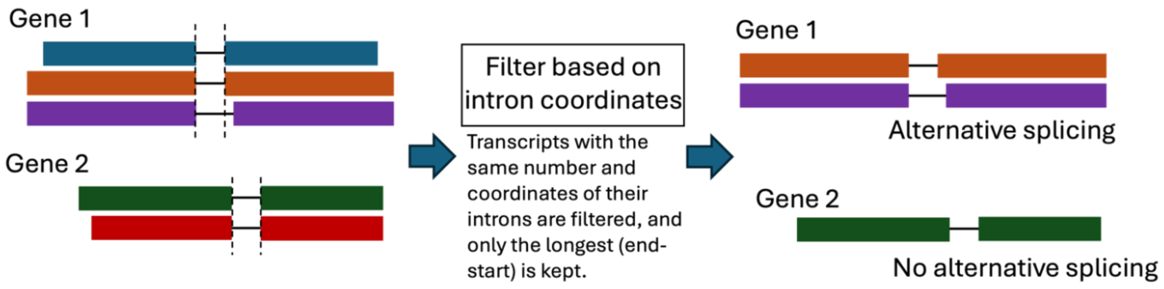

Fig. Assembled transcripts undergo a filtering step, in which transcripts with the same number and coordinates of introns (i.e. identical introns) are identified and only the longest transcript sharing all introns is kept. As a result of this filtering step, the resultant transcripts have different sets of introns.

3. The dispensable chromosome Chr14 of Race 5 is absent in Race 4. How did the dispensable chromosome affect the liftover process?

Response: We thank the reviewer for this question. Indeed, as we previously demonstrated (Zaccaron and Stergiopoulos 2024), *C. fulvum* Race 4 does not possess Chr14. This genomic difference was reflected in our transcriptome assembly results, as (i) we observed that all genes on Chr14 had no assembled transcripts based on the mapped reads from Race 4, which is consistent with the absence of this chromosome, and (2) the liftoff process involved transferring of transcripts only from the 13 core chromosomes to the genome of Race 4. This approach ensured that our analysis accurately represented the genomic and transcriptomic differences between the two races, particularly with respect to the absence of Chr14 in Race 4.

References:

Zaccaron, A. Z., and Stergiopoulos, I. 2024. Analysis of five near-complete genome assemblies of the tomato pathogen *Cladosporium fulvum* uncovers additional accessory chromosomes and structural variations induced by transposable elements effecting the loss of avirulence genes. BMC Biol. 22:25

4. When expression of transcripts was quantified, how did the quantification approach (SALMON) handle the common sequences between isoforms of a gene?

Response: We appreciate the reviewer's inquiry about the reliability of our transcript quantification method. Indeed, quantifying highly similar transcripts can be challenging. We chose Salmon because it is currently one of the most widely used and reliable methods for transcript quantification, particularly for distinguishing between highly similar transcripts. To do so, Salmon employs a probabilistic model that accounts for uncertainty in read assignment, especially for reads mapping to multiple transcripts or isoforms (Patro et al. 2017). This model effectively handles common sequences shared between isoforms.

To demonstrate Salmon's ability to distinguish between similar isoforms and accurately quantify their expression, we conducted a simulated RNAseq experiment in which RNAseq reads were simulated using the R package Polyester v1.39.0 (Frazee et al. 2015). For this experiment, we used all transcripts from genes located on chromosome Chr4 of *C. fulvum* Race 5, which included 2,408 transcripts from 1,396 genes. The

base expression level of all transcripts was set to 20x coverage per transcript, following the Polyester package manual (https://github.com/alyssafrazee/polyester), while specific transcripts from the effector gene *Ecp12* (CLAFUR5_14662) and the predicted transcription factor gene CLAFUR5_04119 were selected for induced expression at specific time points. Transcript quantification was then performed with Salmon V1.10.0 as described in the manuscript, with settings 'Index built with parameter –keepDuplicates' and 'Expression quantified in mapping-based mode (parameters: -l IU, --gcBias, --seqBias)'. The results of this experiment are summarized in the Figures A and B below, which compare the simulated fold change and the resulting expression values estimated by Salmon. As expected, Salmon accurately reported expression peaks for the transcripts that matched their simulated fold change values. This demonstrates Salmon's capability to quantify transcripts with common sequences among isoforms. Overall, this simulation provides strong evidence for the reliability of our transcript quantification method, even for highly similar isoforms. The R code used to simulate the expression of the transcripts and generate the reads is available at https://gist.github.com/alexzaccaron/9429d6b34c82ce0afdf34f43d0af7de2.

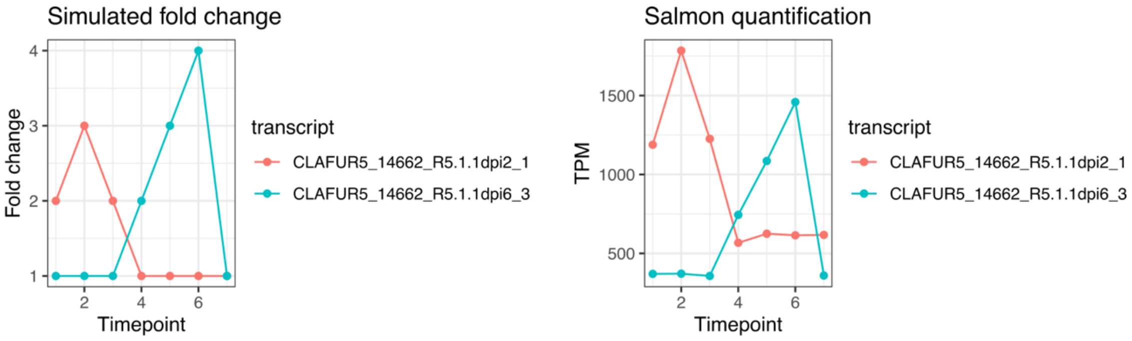

Transcripts aligned

```
CLAFUR5_14662_R5.1.1dpi2_1     ------------------------------------------------------------------------------------------
CLAFUR5_14662_R5.1.1dpi6_3     GAAATAAGCAGTTATAAGACATATAGACCAATATAAATAGTGATAGCACAATGTCCTTTGGGCAACTGTGTCTACTCACAACTCAGCCTT

CLAFUR5_14662_R5.1.1dpi2_1     ------------------ATGCTCTATCTCGCTCTAACGATCGTAGCTTGTCTGCTCTATCCTACCCTCGCTAAGGATAGCACTAGCACG
CLAFUR5_14662_R5.1.1dpi6_3     TTTCTTCTAAGCCTAAAGATGCTCTATCTCGCTCTAACGATCGTAGCTTGTCTGCTCTATCCTACCCTCGCTAAGGATAGCACTAGCACG
                                                 ************************************************************************

CLAFUR5_14662_R5.1.1dpi2_1     GGAAAAGGAGCTAGATGGACTCCAGGCAAAGATGATGCCTTGTGCTATTGCGTTAACAGCCCGCCCGACGTCTCCCAACCGGCCGTAAAT
CLAFUR5_14662_R5.1.1dpi6_3     GGAAAAGGAGCTAGATGGACTCCAGGCAAAGATGATGCCTTGTGCTATTGCGTTAACAGCCCGCCCGACGTCTCCCAACCGGCCGTAAAT
                               ******************************************************************************************

CLAFUR5_14662_R5.1.1dpi2_1     GATAACCAGGCGACTACAAAGGTCTGCCTATCTTACCCGCACGCGCACTCCTTCACGTCTTTACGACATAAGGACGTTCTG------TGT
CLAFUR5_14662_R5.1.1dpi6_3     GATAACCAGGCGACTACAAAGGTCTGCCTATCTTACCCGCACGCGCACTCCTTCACGTCTTTACGACATAAGGACGTTCTGAACAAGTGT
                               *********************************************************************************      ***

CLAFUR5_14662_R5.1.1dpi2_1     ATGGGCAACGAAGATTTTGCCGACTCTGGCCCCGCGTCTAAGGAATTTGCTGATAAGTGTATCGACGTTGGATCAGTTGGGGCTGTCTGT
CLAFUR5_14662_R5.1.1dpi6_3     ATGGGCAACGAAGATTTTGCCGACTCTGGCCCCGCGTCTAAGGAATTTGCTGATAAGTGTATCGACGTTGGATCAGTTGGGGCTGTCTGT
                               ******************************************************************************************

CLAFUR5_14662_R5.1.1dpi2_1     TGCCCGCAGAAGACACAAGGCTTGCCTGATGATGGGACGGAGTGTTTTGTTGCGTTCCCGTCGTGA------------------------
CLAFUR5_14662_R5.1.1dpi6_3     TGCCCGCAGAAGACACAAGGCTTGCCTGATGATGGGACGGAGTGTTTTGTTGCGTTCCCGTCGTGAAGACTGGTGTAGACTTATCTCCGT
                               ******************************************************************

CLAFUR5_14662_R5.1.1dpi2_1     ------------------------------------------------------------------------------------------
CLAFUR5_14662_R5.1.1dpi6_3     GCTTCCGCAATTCTGTCGATTCTTGGCACTGGGTTGTAATAGGTTGAGGTGTTCTACGTGACATCTTAGAGTCTATTCGTTTTACTCGTT

CLAFUR5_14662_R5.1.1dpi2_1     -------------------------------------
CLAFUR5_14662_R5.1.1dpi6_3     GCAAGCCCCACGCCAAAGGCGTGTCGTCCGGCTCAAC
```

Fig A. Quantification of simulated expression of transcripts from the effector gene *Ecp12* (CLAFUR5_14662). The isoforms of *Ecp12* were induced either early or late in the simulated time points. The quantification of the transcripts performed by Salmon based on the simulated reads showed matching peaks of expression compared to the simulated fold change values. Alignment of the transcript sequences is shown below the expression plots.

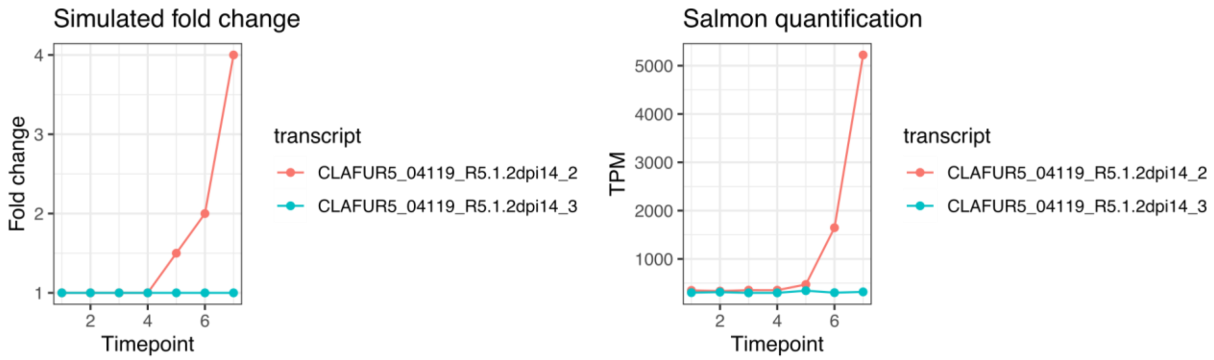

Transcripts aligned

```
CLAFUR5_04119_R5.1.2dpi14_2    ---------------ACAGCATGTAACATTCCTCCAGCACTTGCTATCAGCATCATGACGACGGAATTTAGCAGCCGTCCCTGGCAGTATAAAAGAC
CLAFUR5_04119_R5.1.2dpi14_3    GGCGTCCATGCATGCACAGCATGTAACATTCCTCCAGCACTTGCTATCAGCATCATGACGACGGAATTTAGCAGCCGTCCCTGGCAGTATAAAAGAC
                                              **********************************************************************************

CLAFUR5_04119_R5.1.2dpi14_2    AACGATTCGCCACCTCAACTGCCGACACAAGACATTCGATTCGATCAGTCATATGAACATGACGATGACATTGACACCGCAGGAAGAGGCCGAATTG
CLAFUR5_04119_R5.1.2dpi14_3    AACGATTCGCCACCTCAACTGCCGACACAAGACATTCGATTCGATCAGTCATATGAACATGACGATGACATTGACACCGCAGGAAGAGGCCGAATTG
                               *************************************************************************************************

CLAFUR5_04119_R5.1.2dpi14_2    CAATGGCTGCTGCAAGGAATGCATTCCGTGCACAGCCAACAGAATCCCATCCGAGCTGACATTGTGCACGATTTGGAGCACAATTCCGCGACAGCCG
CLAFUR5_04119_R5.1.2dpi14_3    CAATGGCTGCTGCAAGGAATGCATTCCGTGCACAGCCAACAGAATCCCATCCGAGCTGACATTGTGCACGATTTGGAGCACAATTCCGCGACAGCCG
                               *************************************************************************************************

CLAFUR5_04119_R5.1.2dpi14_2    ACCACTCGGCAGCCGCGCCGTGGCCCTCCTCGCCCTCGACCTCGCCTCCGAATATCAGCTACAACGAGGCAGTGACGAAAGTGCTTCGACGAAAGCA
CLAFUR5_04119_R5.1.2dpi14_3    ACCACTCGGCAGCCGCGCCGTGGCCCTCCTCGCCCTCGACCTCGCCTCCGAATATCAGCTACAACGAGGCAGTGACGAAAGTGCTTCGACGAAAGCA
                               *************************************************************************************************

CLAFUR5_04119_R5.1.2dpi14_2    GCAGAATCGTGAAGCCCAACGAAAGTGCCGAGACCGTCAGAAAGACATGATCGCTGGGTTGAACGAGCGGTTGCGATTGAGCAATGGCGCGAACAGC
CLAFUR5_04119_R5.1.2dpi14_3    GCAGAATCGTGAAGCCCAACGAAAGTGCCGAGACCGTCAGAAAGACATGATCGCTGGGTTGAACGAGCGGTTGCGATTGAGCAATGGCGCGAACAGC
                               *************************************************************************************************

CLAFUR5_04119_R5.1.2dpi14_2    GAGCTACAAGCCAAAGTCCGAGAATTGAGCGCAGAATGTGGTCGACTTCGTGCTGAGAATGCTGTACACATTGAACTGCTACAAAGCTTTTGGTCAT
CLAFUR5_04119_R5.1.2dpi14_3    GAGCTACAAGCCAAAGTCCGAGAATTGAGCGCAGAATGTGGTCGACTTCGTGCTGAGAATGCTGTACACATTGAACTGCTACAAAGCTTTTGGTCAT
                               *************************************************************************************************

CLAFUR5_04119_R5.1.2dpi14_2    CTGGAGCATGCATCTGCGAGAATGGTGCCGGTGCCTTTGACGCAGTATACCCTCCTATAACCAATACGGCAAATTGGAGTGCTCAGGCCGCATCAAC
CLAFUR5_04119_R5.1.2dpi14_3    CTGGAGCATGCATCTGCGAGAATGGTGCCGGTGCCTTTGACGCAGTATACCCTCCTATAACCAATACGGCAAATTGGAGTGCTCAGGCCGCATCAAC
                               *************************************************************************************************

CLAFUR5_04119_R5.1.2dpi14_2    AAGAGCAACGCGGCTTTGACCAGGACATTCATCGTAGAAGGAGCCTG-----AGGACTTGCAAGCATTTTTGACCATCCACTACAACCAATGCACCC
CLAFUR5_04119_R5.1.2dpi14_3    AAGAGCAACGCGGCTTTGACCAGGACATTCATCGTAGAAGGAGCCTGATCTAGGGACTTGCAAGCATTTTTGACCATCCACTACAACCAATGCACCC
                               ***********************************************      ********************************************

CLAFUR5_04119_R5.1.2dpi14_2    TCTAAAGTAGTATCTGTGCCAGGACCGAGACCTCAGTCTCTTTGCACCCCGACCTTGCCAACCTTTCTCAAGCCGACGCCTGCAATGAC
CLAFUR5_04119_R5.1.2dpi14_3    TCTAAAGTAGTATCTGTGCCAGGACCGAGACCTCAGTCTCTTTGCACCCCGACCTTGCCAACCTTTCTCAAGCCGACG-----------
                               ******************************************************************************
```

Fig B. Quantification of simulated expression of transcripts from the predicted transcription factor gene CLAFUR5_04119. The expression of the isoforms of the gene CLAFUR5_04119 was induced at late time points in the simulated time course. The quantification of the transcripts performed by Salmon based on the simulated reads showed a matching peak of expression compared to the simulated fold change values. Alignment of the transcript sequences is shown below the expression plots.

References

Frazee, A. C., Jaffe, A. E., Langmead, B., and Leek, J. T. 2015. Polyester: simulating RNA-seq datasets with differential transcript expression. Bioinformatics. 31:2778–2784

Patro, R., Duggal, G., Love, M. I., Irizarry, R. A., and Kingsford, C. 2017. Salmon provides fast and bias-aware quantification of transcript expression. Nat. Methods. 14:417–419
